# Health and Economic Impacts Assessment of O_3_ Exposure in Mexico

**DOI:** 10.3390/ijerph182111646

**Published:** 2021-11-05

**Authors:** José Luis Texcalac-Sangrador, Magali Hurtado-Díaz, Eunice Elizabeth Félix-Arellano, Carlos Manuel Guerrero-López, Horacio Riojas-Rodríguez

**Affiliations:** 1 Environmental Health Department, National Institute of Public Health, Cuernavaca 62100, Mexico; jtexcalac@insp.mx (J.L.T.-S.); mhurtado@insp.mx (M.H.-D.); eunice_1734@hotmail.com (E.E.F.-A.); 2 Independent Researcher, Mexico City 03303, Mexico; mces.carlos.guerrero@gmail.com

**Keywords:** SOMO35, health impact assessment, economic impacts, air pollution

## Abstract

Health effects related to exposure to air pollution such as ozone (O_3_) have been documented. The World Health Organization has recommended the use of the Sum of O_3_ Means Over 35 ppb (SOMO35) to perform Health Impact Assessments (HIA) for long-term exposure to O_3_. We estimated the avoidable mortality associated with long-term exposure to tropospheric O_3_ in 14 cities in Mexico using information for 2015. The economic valuation of avoidable deaths related to SOMO35 exposure was performed using the willingness to pay (WTP) and human capital (HC) approaches. We estimated that 627 deaths (95% uncertainty interval (UI): 227–1051) from respiratory diseases associated with the exposure to O_3_ would have been avoided in people over 30 years in the study area, which confirms the public health impacts of ambient air pollution. The avoidable deaths account for almost 1400 million USD under the WTP approach, whilst the HC method yielded a lost productivity estimate of 29.7 million USD due to premature deaths. Our findings represent the first evidence of the health impacts of O_3_ exposure in Mexico, using SOMO35 metrics.

## 1. Introduction

Air pollution is considered to be one of the main environmental risks to health around the world [1]. Several studies have documented the association between harmful effects on human health and exposure to air pollutants [2,3], among them ozone (O_3_).

O_3_ is a photochemical oxidant that may cause oxidative damage to the cells and lining fluids of airways, thus inducing immune-inflammatory responses and potentially causing adverse health effects [4]. Short-term effects, especially during the warm season, include hospital respiratory admissions [5], premature deaths [6], cardiovascular and respiratory mortality [7], as well as adverse effects on the central nervous system [8]. On the other hand, evidence of long-term effects includes increased asthma incidence [9]; reduced lung function and lung growth [10]; and an increased risk of low birth weight [11], lung cancer [12], and premature mortality [13].

Epidemiological studies provide evidence to support local governments and international organizations in implementing public policies which aim to reduce adverse health effects [14]. The results of these studies have been used to conduct Health Impact Assessments (HIA) worldwide. This type of assessment has been consolidated as a methodological tool to assist decision-makers in evaluating, quantitatively and qualitatively, how any policy, program, or project may affect human health (e.g., the impact of interventions on controlling air quality and reducing effects on human health) [15]. In addition, economic valuations of the adverse health effects of air pollution facilitate awareness of some of the consequences of public policies or the lack thereof. This, in turn, helps decision-makers to formulate and implement more consistent and explicitly informed policies, since putting health effects in terms of money makes it possible to assess interventions separately and to compare the costs and benefits of public policies that aim to reduce air pollution.

To perform HIAs of long-term exposure to O_3_, the World Health Organization (WHO) has recommended the use of the Sum of O_3_ Means Over 35 ppb (SOMO35) [16,17], which is defined as the annual sum of the daily maximum of the 8 h moving average over 35 ppb. This indicator emerged due to uncertainties in O_3_ studies regarding the effects of long-term exposure and the slope of the concentration–response function at lower concentrations [18], and it is a useful chronic exposure indicator for quantifying health impacts [19].

Several HIAs have been performed worldwide [20,21,22] using SOMO35 [19,23,24]; however, in Mexico, HIAs have focused on particulate matter [25]. The lack of information concerning SOMO35 health estimates and their associated costs has impeded the provision of information on the contribution of O_3_ exposure in excess of 35 ppb.

Although efforts have been made in Mexico to reduce O_3_ air pollution during recent years, only 21 out of 149 stations met the O_3_ national ambient air quality standard in 2018, and 74 stations exceeded the 1 h (0.095 ppm) and 8 h (0.07 ppm) limits. For example, the Mexico City Metropolitan Area (MCMA) exceeded the 1 h standard on 59.7% of days, followed by the Metropolitan Areas of Guadalajara (32.6%) and Monterrey (9.9%) [26].

The aim of this study was to assess the health and economic impacts of long-term O_3_ exposure in 14 Mexican cities for 2015. To our knowledge, this is the first HIA study using the SOMO35 metrics of O_3_ in Mexico. 

## 2. Materials and Methods

An HIA was performed to estimate the avoidable mortality associated with long-term exposure to tropospheric O_3_ in 14 cities in Mexico using information from 2015 and following the WHO-HIA methodology [25,27,28,29]. Our analysis and estimates were carried out at the municipal level and aggregated by metropolitan or conurbation area, as defined by the National Urban System (SUN is its acronym in Spanish). Data processing, geographic layers, and estimates were performed using R version 3.3 (R Core Team, Vienna, Austria) and the RStudio Desktop version 1.1.463 (RStudio Team, Boston, MA, USA) software.

### 2.1. Air Pollution Data

Hourly O_3_ concentrations were obtained from the automatic monitoring stations of the National System of Air Quality Information (SINAICA is its acronym in Spanish) to estimate the annual sum of the daily maximum of the 8 h moving average over 35 ppb (SOMO35); this metric uses the concentration of 35 ppb as the O_3_ cut-off value. The daily maximum of the moving 8 h average O_3_ concentration was selected from each station when it met a minimum of 75% completeness of hourly data (18 h per day) for a minimum of 75% of the days of the year.

For each monitoring station, the SOMO35 indicator was estimated according to the following equations [17]:

(1) $$SOMO35_{uncorrected}=\overset{d=N_{y}}{\underset{d=1}{\sum }}\max(A_{8}^{d}-35ppb,0)$$

Then:

(2) $$SOMO35=SOMO35_{uncorrected}\times \frac{N_{y}}{N_{Valid}}$$

where *SOMO35_uncorrected_* is the uncorrected *SOMO35*, $A_{8}^{d}$.$\;A_{8}^{d}$ is the maximum daily 8 h running average of O_3_ on day *d*, *N_y_* is the total number of days for a year, and *N_valid_* is the number of days with available valid data. The hourly concentrations were treated accordingly to calculate the proper averages. 

### 2.2. Cities Selection and Population Exposure

Each metropolitan or conurbation area comprises a varied number of municipalities, and these, in turn, comprise several basic geostatistical areas (for which AGEB is the acronym in Spanish). An AGEB is defined as a geographic space consisting of a set of blocks, generally ranging from 1 to 50 in number, delimited by streets, avenues, or any other features easily identifiable on the land and whose land use is mainly housing, industrial, services, or commercial. We selected only those municipalities with the spatial representativeness of O_3_ monitoring data. For this purpose, we selected the municipalities in which more than 50% of their AGEBs were located at intersections between the 5 km circular buffers that surrounded each of the O_3_ monitors.

The SOMO35 concentration was estimated at the municipal level through a spatial analysis and interpolation processes using geographic information layers in a shapefile format. First, monitoring stations were allocated according to their geographic coordinates and a 5 km circular buffer was created around each site. Second, intersection areas between the 5 km buffers were identified and the AGEB centroids located within them were selected; then, their SOMO35 concentration was estimated using square-weighted IDW. This step was replicated using 10 km buffer intersection areas, excluding the previously estimated centroids. Next, the non-estimated centroids located within each 10 km buffer intersection area were identified and assigned with the concentration from the monitoring station. Finally, the concentrations of the centroids that were not estimated in the previous steps were estimated using IDW with a weighting of 1. The final concentration of SOMO35 at the municipal level was calculated by adding and averaging the AGEB values. The concentration for each metropolitan area and/or conurbation was obtained by adding together the average concentrations obtained on the municipal scale.

### 2.3. Concentration–Response Function

We used the Concentration–Response Function (CRF) recommended by the WHO Regional Office for Europe (WHO-Europe); this was based on an exhaustive review of scientific evidence of the health effects of air pollution. The selected CRF indicates that an increase of 10 μg/m^3^ for the SOMO35 indicator increases the risk of death from respiratory causes (ICD-10 J00-J98) in people over 30 years of age by 1.014 (95% CI 1.005–1.024) [30].

### 2.4. Health and Demographic Data

Population and mortality data for 2015, at the municipality and AGEB level, organized by age group, were provided by the National Institute of Statistics and Geography (for which INEGI is the acronym in Spanish). Mortality data were aggregated by cause according to the CRF selected by municipality.

### 2.5. Avoidable Deaths

To estimate avoidable deaths from respiratory diseases associated with long-term O_3_ exposure, first the CRF was standardized to a unit of change using the following formula:

(3) $$CRF=e^{ln\left(RR\right)/\Delta C}$$

where *RR* corresponds to the *RR* of the *CRF* and ∆**C** corresponds to the *RR* change unit. Then, the next equation was used:

(4) $$Avoidable\;deaths=total\;deaths-\frac{total\;deaths}{e^{\left([\ln CRF]\;*\;SOMO35\right)}}$$

where *Avoidable deaths* corresponds to the deaths from respiratory diseases in the given age group, *SOMO35* is the *SOMO35* concentration, and *CRF* is the *RR* transformed into a unit of change.

### 2.6. Economic Assessment

The economic valuation of avoidable deaths from respiratory diseases related to SOMO35 exposure was performed using the willingness to pay (WTP) and human capital (HC) approaches. First, we estimated the value of a statistical life (*VSL*) by applying the following equation, as suggested by the Organization for Economic Cooperation and Development (*OECD*) [31].

(5) $$VSL_{S}^{2015}=VSL_{OECD}^{2015}*{\left(\frac{Y_{S}^{2015}}{Y_{OECD}^{2015}}\right)}^{\beta }$$

where *S* is the city; *VSL* in *OECD* in 2015 is estimated to be 3.59 million USD after adjusting a previous *VSL* estimate [31]. for inflation [32]; *Y* is the gross domestic product (GDP) per capita in city *S* in 2015 gathered from the National Institute of Statistics and Geography [33]; and *β* is the income elasticity of 0.9 for middle-income countries [31]. For sensitivity purposes, we also calculated the *VSL* using *β =* 1.5 (lower value) and *β* = 0.5 (upper value for VSL). When a city included municipalities of more than 1 state, we calculated the GDP per capita weighting by the relative population within the city.

The HC perspective relies on the assumption that the flow of future annual productivities is lost because of premature death. We estimated the productivity loss at every age (*Yd*) of death in every city as the sum of future expected productivities obtained from the National Survey on Occupation and Employment (for which ENOE is the acronym in Spanish) [34]. ENOE included more than 1.15 million individual observations in the 4 quarters of 2015 at the national level. We used the Mincer equation to estimate expected annual productivity (*Y_age_*), adjusting by sex, age, the quarter of the year, age square, social security, schooling, and city among individuals with positive earnings, assuming 240 working 8 h days per year. To calculate the present values of future monetary figures, we discounted the expected productivities at a 3% annual rate:

(6) $$Y_{d}=\left(\overset{LE}{\underset{t=30}{\sum }}\frac{Y_{age}}{{\left(1-r\right)}^{t-30}}\right)$$

where *LE* = life expectancy = 76 years, *Y_age_* = annual productivity at a certain age, *t* = age at death, and *r* = discount rate = 0.03. For deaths at ages older than 76 years, we assumed the lost productivity at 76 years old. We calculated the discounted expected productivities using 95% lower and upper confidence interval values to provide uncertainty analyses. To calculate the economic costs associated with mortality by O_3_, we multiplied the number of avoidable deaths within each city by the corresponding VSL and lost productivity at every age at death. We considered a purchasing power parity (PPP) of 8.33 MXN = 1 USD for both approaches [35].

## 3. Results

### 3.1. Study Area

**Figure 1:**
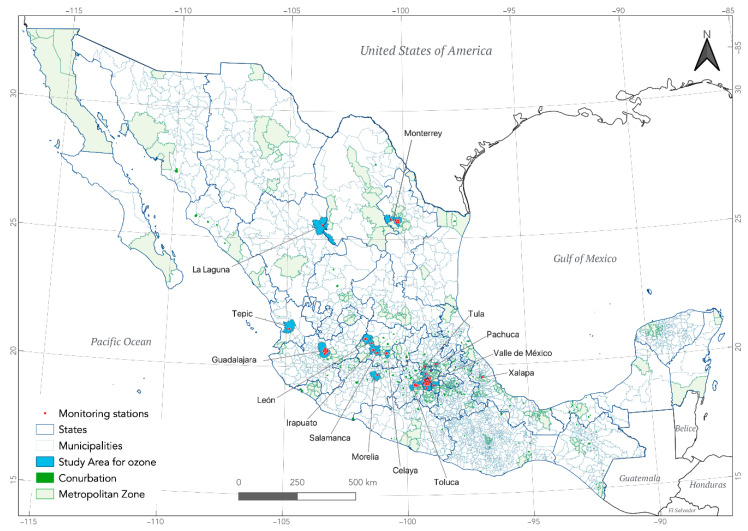
Study areas.

The study area consisted of 67 municipalities distributed across 14 cities which, according to the SUN, correspond to the conurbations of Irapuato and Salamanca in the state of Guanajuato, and 12 metropolitan areas, all of them distributed in 11 states of the country; this area includes more than 33 million people (Figure 1). 

Figure 1 shows the conurbations and metropolitan areas that, according to Mexican regulation, should monitor the air quality. It was not possible to include all of them in the analysis due to a lack of information or insufficient data in the monitoring stations.

Table 1 shows the number of municipalities included in the analysis, their cities, and their population. The total population of the 14 cities was 33,526,996 inhabitants; the Valle de Mexico, Guadalajara, and Monterrey had the largest populations of the cities included in the analysis. 

### 3.2. Estimates of Exposure to O_3_

For 2015, it was possible to collect the information from 117 monitoring stations; however, only 59 (50.4%) met the criterion of a 75% sufficiency of data. Regarding O_3_ concentration, the municipalities of the metropolitan area of the Valle de Mexico, León, Celaya, and Irapuato presented the highest concentration considering the SOMO35 indicator (Table 1).

### 3.3. Economic and Health Impacts (Avoidable Deaths)

We estimated that 627 (9% UI: 227–1051) deaths from respiratory diseases associated with exposure to O_3_ would be avoided in people over 30 years in the study area—this represents 4% of the total deaths in the study area for the same cause and age group. The metropolitan area of the Valle de Mexico had the greatest impact, with 461 avoidable deaths (Table 2); Mexico City alone accounts for 66% of this total and exceeds the metropolitan area of Guadalajara, which occupies the second position.

Avoidable deaths account for almost 1400 million USD under the WTP approach, whilst the HC methods yielded a lost productivity estimate of 29.7 million USD caused by premature deaths and the central (point) estimates. Table S1 shows the productivity lost by city and age.

## 4. Discussion

This study represents the first quantification of the health impacts attributable to long-term O_3_ exposure in Mexican cities using the indicator recommended by the World Health Organization (SOMO35). The avoidable deaths for respiratory diseases associated with O_3_ exposure amount to 627 deaths in the study area, which confirms the public health impacts of ambient air pollution.

SOMO35 levels vary between metropolitan areas, but the MCMA, the most populated city, stands out with the highest concentration and contribution to the estimates of avoidable deaths. It is important to mention that the meteorological conditions in this region, along with the presence of precursor emissions, promote the formation of O_3_ during half of the year. However, although some cities have relatively high O_3_ concentrations, such as Salamanca, they did not represent a high contribution to avoidable deaths.

So far, the metrics used to evaluate the health effects associated with O_3_ exposure are primarily acute (1 h or 8 h moving averages), and studies of long-term exposure to O_3_ are based on the annual or warm season average of daily 8 h maximum concentrations [13,36]. This approach implies the use of higher concentrations compared to SOMO35, which is an indicator of the accumulated O_3_ concentration in excess of 35 ppb (70 μg/m^3^) during the whole year. The difference between these approaches represents a significant element in estimating deaths attributable to O_3_ exposure. Moreover, SOMO35 is a useful indicator for quantifying the health impacts of long-term exposure to O_3_ and a reliable procedure for estimating mortality attributable to O_3_ [37]; it has been used by the international scientific community [19,38], and therefore our results are likely to be highly reliable.

For 2015, the GBD estimated more than 1991 deaths (888–3247) attributable to environmental exposure to O_3_ for Mexico while we estimated 627 deaths. There are important differences between both studies such as the study area, the metrics of the exposure, and the causes of mortality. Our study is based on measurements from monitoring stations in 14 cities, uses the SOMO35 metric, and considers only deaths from respiratory diseases. Meanwhile, the GBD is based on chemical transport models for the entire country, uses the running 3-month (summer) mean of O_3_ concentration (of daily 1 h maximum values) as the metric of exposure, and selects the maximum of these values for each 0.1 × 0.1° (∼11 km × 11 km) over 1 year. Furthermore, the GDB considers only Chronic Obstructive Pulmonary Disease (COPD) as a specific cause for analysis. The GBD results are higher because of these differences.

In Mexico, a previous study called the “Central Region Study” quantified the avoidable deaths attributable to O_3_ exposure using a different approach [39]. It included seven states, with a population of 25 million inhabitants, and most of the cities with high O_3_ concentrations in Mexico. This study reports a total of 1067 avoidable deaths in contrast to our estimate of 627 deaths. Once more, the metrics to evaluate O_3_ exposure play an important role. The “Central Region Study” uses an annual O_3_ average concentration which is calculated from the maximum 8-h moving O_3_ averages, and a counterfactual scenario of 50 ppb. We used the SOMO35 indicator which is an optimal alternative to estimate chronic exposure; it uses the information from every day with moderate and constantly high O_3_ concentrations and not only the days with very high concentrations. In general, studies using different approaches from SOMO35 tend to overestimate the attributable deaths. 

To contrast our results with different cut points, we used SOMO50 and SOMO0. The deaths attributable to respiratory diseases associated with exposure to O_3_ were estimated using the SOMO50 (exceeds of 50 ppb). The avoidable deaths attributable to O_3_ exposure decreased from 627 to 275 deaths, which is almost a 50% difference. The opposite extreme implies estimating avoidable deaths without considering a background concentration and without removing any excess, which is known as SOMO0. Under this consideration, the avoidable deaths attributable to O_3_ exposure would increase to 994. This difference reveals the importance of selecting the exposure metric and its counterfactual value when generating impact estimates. In September 2021, the WHO released new air quality guidelines (WHO-AQG), specifying 60 ug/m^3^ (30 ppb) as the limit value for O_3_ long-term exposure; using this concentration as a cut-off point, we observe that 782 deaths could have been avoided in 2015. It means that health impacts of this cut-off would be greater since, on average, 150 additional deaths would be avoided, compared to the estimates using 70 ug/m^3^ as the cut-off point.

As already mentioned, the SOMO35 indicator is calculated from the selection of the highest concentration of the moving averages of 8 h of each day; this value may vary depending on the weather season and other factors.

Our estimates of respiratory diseases associated with the exposure to O_3_ may be added to those already calculated for PM_2.5_ exposure. As Trejo et al. pointed out [25], these respirable particles generate more than 14,600 avoidable deaths per year considering only those cities with sufficient data to perform the evaluation. Unlike our study, Trejo et al. used information from 15 cities with a larger population, and in which the set of diseases related to exposure to PM_2.5_ was larger, the CRF was different, and the age groups were wider. However, both studies are complementary and are useful for pointing out the mortality attributable to air pollution in Mexico.

With respect to economic valuation, we offer two types of estimates that rely on different theoretical backgrounds, and thus produce estimates of different orders of magnitude. Usually, VSL estimates present a wide range of values depending on assumptions and methods, as discussed elsewhere [25]. Although the ideal way to calculate a VSL in Mexico is to use reliable stated preferences studies concerning money–risk tradeoffs, no large, representative surveys exist in Mexico on that subject; thus, this is one major limitation of this study. However, our estimates are based on the systematic review and methods outlined by the OECD in order to transfer a base VSL to Mexico and in order to calculate specific VSLs for the cities included in this study, taking into account heterogeneity in GDP per capita among them, as suggested by Hammit [40]. We estimated a VSL of 1.76 million USD at the national level (this estimate is not included in Table 2, but is used here for comparison purposes), which is not far from the 1.671 million USD calculated by Viscusi et al. [41] for Mexico in 2015. The same study found that the average VSL for upper middle-income countries was 1.2 million USD. On the other hand, the HC approach represents lost productivity caused by premature death. Our calculations were performed using the standard methods and the best available sources of information on employment and wages in Mexico. The costs of mortality, which we calculated using the WTP approach, accounts for 0.113% of the GDP in 2015 in the States where the cities analyzed are located. Among policy analysts, the VSL is generally considered to be an economically adequate measure of the benefit individuals receive from enhancements to their health or safety [42]. Although the estimates made using the HC approach seem negligible compared with VSL estimates, we argue that this information provides further valuable evidence of the economic consequences of air pollution. A previous study performed in Mexico on preventable deaths related to exposure to PM_2.5_ also produced estimates for the VSL in Mexico in 2015 [25]. The main difference of the point estimate in that study (1.643 million USD) compared to our new estimate (1.76 million USD) is that we used updated GDP per capita data by state. Moreover, in the current study, we were able to estimate the cost of avoidable deaths using the WTP approach by calculating the specific VSL for every city included, and therefore this is one of the main improvements of this study.

Limitations: our estimates are based on the measurements from monitoring stations whose operation occurs irregularly over time. This limits the number of monitoring stations with available information to calculate SOMO35, as well as the number of cities and metropolitan areas that can be studied. If there were better monitoring coverage and consistency in measurements, the estimated impacts would be greater. However, the analysis includes the metropolitan areas that historically have had the highest levels of O_3_.

Recommendations: an estimate of the burden attributable to O_3_ exposure morbidity in Mexican cities is recommended. Although we know that the greatest burden occurs due to avoidable mortality, the increase in signs, symptoms, and respiratory diseases can rise significantly in these cities during the warm season. The results would be useful for local air quality improvement programs.

## 5. Conclusions

Our findings represent the first evidence of the health impacts of O_3_ exposure using the SOMO35 metrics in Mexico. The implementation of the HIA method using SOMO35 allows us to quantify the magnitude of avoidable deaths from respiratory diseases associated with long-term exposure to O_3_ in the Mexican population and its associated economic costs, considering 2015 as a reference. Likewise, our study evidences this impact at different scales within the geostatistical and urban frameworks of Mexico, constituting a support document for decision making at various scales of the territory.

## Figures and Tables

**Table 1:** Number of municipalities and population in the cities included in the study, 2015.

State	City	Population	Population > 30 Years	Mortality Rate ^a^	Mun	SOMO35 by Mun Mean (Min–Max)
Nuevo León	Monterrey *	3,615,075	1,810,793	85.2	7	7.82 (5.74–13.4)
Coahuila Durango	La Laguna *	1,174,885	556,665	77.5	3	1.5
Nayarit	Tepic *	471,026	217,436	76.7	2	6.4
Jalisco	Guadalajara *	4,725,603	2,194,989	90.6	6	7.63 (5.98–9.16)
Guanajuato	León *	1,768,193	771,130	77.5	2	16.1 (16.1–16.6)
Guanajuato	Irapuato ^†^	574,344	251,291	91.0	1	19.4
Guanajuato	Salamanca ^†^	273,271	134,162	74.1	1	16.3
Guanajuato	Celaya *	494,304	227,157	55.9	1	19.1
Morelia	Morelia *	784,776	371,420	115.8	1	9.2
Hidalgo	Pachuca *	427,551	210,422	80.9	2	15.5
Hidalgo	Tula *	68,247	32,222	77.8	2	12.2
Estado de Mexico	Toluca *	1,512,455	712,063	86.0	5	8.22 (7.46–9.71)
Mexico City Estado de Mexico	Valle de Mexico *	17,156,425	9,157,798	90.3	33	20.8 (8.9–29.0)
Veracruz	Xalapa *	480,841	241,693	81.9	1	4.5
Total		33,526,996	16,889,241	85.2	67	14.8 (1.5–29.0)

^a^: Mortality rate from respiratory causes; *: metropolitan area; ^†^: conurbation; Mun: municipalities; SOMO35 concentration in ppb; min: minimum; max: maximum.

**Table 2:** Annual avoidable deaths from respiratory diseases associated with exposure to O_3_, 2015 ^a^.

City	Avoidable Deaths (95% UI)	Economic Valuation ^b^ (Min–Max)	Lost Productivity (USD)
Irapuato	11 (4–18)	$15.9 (8.7–23.8)	$311,673.4 (228,810.7–394,536.1)
Salamanca	4 (2–7)	$5.8 (3.2–8.7)	$142,685.1 (113,212–173,821.5)
La Laguna	1 (0–3)	$2.0 (1.4–2.5)	$78,669.9 (70,110.1–87,229.6)
Valle de Mexico	461 (166–767)	$1106.3 (962.4–1278.7)	$22,395,473.2 (21,018,939–23,772,007.4)
Celaya	9 (3–15)	$13.0 (7.1–19.5)	$357,668.0 (264,351.7–450,984.3)
León	30 (11–51)	$43.4 (23.7–65.0)	$1,103,163.9 (1,008,491.8–1,197,836.1)
Pachuca	6 (3–10)	$7.3 (3.5–11.8)	$315,349.6 (286,099.3–344,599.8)
Tula	0	0	0
Guadalajara	50 (19–86)	$88.2 (54.9–120.9)	$2,235,794.5 (2,107,509.1–2,364,079.9)
Toluca	12 (4–20)	$14.0 (6.6–23.1)	$475,383.0 (424,168.1–526,597.9)
Morelia	7 (2–11)	$7.6 (3.4–12.9)	$257,349.4 (234,074.5–280,624.4)
Tepic	3 (1–6)	$3.7 (1.8–6.0)	$96,211.8 (87,603.2–104,820.4)
Monterrey	31 (11–53)	$88.2 (75.6–97.8)	$1,854,470.6 (1,761,086.9–1,947,854.3)
Xalapa	2 (1–4)	$2.5 (1.2–4.0)	$62,171.7 (43,690.6–82,601.2)
Total	627 (227–1051)	$1397.8 (1153.6–1674.6)	$29,686,064.1 (27,648,147–31,727,592.9)

^a^: Population ≥ 30 years of age; ^b^: economic valuation of avoidable deaths (WTP approach, millions USD); USD: United States Dollars; min: minimum; max: maximum: 95% UI: 95% uncertainty interval. Calculations were made using point estimates of avoidable deaths.

## Data Availability

The data presented in this study are openly available in Harvard Dataverse at (https://doi.org/10.7910/DVN/PXVSHA (accessed on 21 October 2021).

## Supplementary Materials

The following are available online at https://www.mdpi.com/article/10.3390/ijerph182111646/s1, Table S1: Productivity lost (Yd).
