# Supplementary material for: Health and Economic Impacts Assessment of O3 Exposure in Mexico
Source: Int J Environ Res Public Health. 2021 Nov 5;18(21):11646. doi: 10.3390/ijerph182111646 (PMC8583463; doi:10.3390/ijerph182111646)
Supplement: Supplementary file 1 [file ijerph-18-11646-s001.zip › ijerph-1345224-supplementary.pdf]

Supplementary table 1: Productivity lost (Yd)

City ID	City name	Age	Avoidable deaths from respiratory causes mean estimate	Lower value, lost productivity	Center value, lost productivity	Upper value, lost productivity	Lost productivity by age, lower value	Lost productivity by age, center value	Lost productivity by age, upper value
C11.05	Irapuato	30	0.051591601	166740.843	197306.8728	227872.9029	8602.427017	10179.37743	11756.32785
C11.05	Irapuato	34	0.051591601	158521.0723	189021.9788	219522.8855	8178.355889	9751.946481	11325.53708
C11.05	Irapuato	35	0.051591601	156268.2787	186819.1459	217370.0133	8062.130657	9638.298804	11214.46696
C11.05	Irapuato	38	0.051591601	148903.4821	178803.7249	208703.9665	7682.169014	9224.770402	10767.37173
C11.05	Irapuato	41	0.103183202	146790.962	177456.6962	208122.4293	15146.36143	18310.55008	21474.7386
C11.05	Irapuato	45	0.051591601	133082.4714	164133.0148	195183.553	6865.937742	8467.884984	10069.83196
C11.05	Irapuato	46	0.051591601	129712.9729	160532.8027	191352.6271	6692.099923	8282.144278	9872.188355
C11.05	Irapuato	47	0.051591601	125681.0568	156398.7016	187116.3408	6484.086913	8068.859383	9653.631565
C11.05	Irapuato	49	0.051591601	116206.9652	145792.3524	175377.7337	5995.303364	7521.66085	9048.018033
C11.05	Irapuato	50	0.051591601	112418.9636	141702.8317	170986.6949	5799.874299	7310.675929	8821.47731
C11.05	Irapuato	55	0.051591601	91254.2585	118180.5382	145106.8163	4707.953279	6097.123154	7486.292944
C11.05	Irapuato	56	0.051591601	86824.15133	113412.3778	140000.6025	4479.396959	5851.126124	7222.855201
C11.05	Irapuato	57	0.051591601	83063.00922	108462.4999	133861.9887	4285.353616	5595.753998	6906.15429
C11.05	Irapuato	58	0.051591601	79245.16612	103589.0483	127932.93	4088.384979	5344.324833	6600.264657
C11.05	Irapuato	59	0.154774803	76941.14504	99740.70665	122540.2677	11908.55053	15437.34818	18966.14573
C11.05	Irapuato	60	0.154774803	72907.6969	94999.35059	117091.0037	11284.27439	14703.50573	18122.73697
C11.05	Irapuato	61	0.103183202	69426.44885	91094.80113	112763.154	7163.643274	9399.453237	11635.26326
C11.05	Irapuato	62	0.206366403	63975.65532	84019.84164	104064.0298	13202.42589	17338.87253	21475.31956
C11.05	Irapuato	63	0.051591601	59911.99009	79483.69333	99055.39973	3090.955478	4100.690979	5110.426644
C11.05	Irapuato	64	0.103183202	54329.94749	72490.40446	90650.86469	5605.937929	7479.792023	9353.646454

City ID	City name	Age	Avoidable deaths from respiratory causes mean estimate	Lower value, lost productivity	Center value, lost productivity	Upper value, lost productivity	Lost productivity by age, lower value	Lost productivity by age, center value	Lost productivity by age, upper value
C11.05	Irapuato	65	0.206366403	46289.20553	63653.93676	81018.67012	9552.53686	13136.03399	16719.53156
C11.05	Irapuato	66	0.051591601	42821.37114	59151.89997	75482.42974	2209.223087	3051.741212	3894.259386
C11.05	Irapuato	67	0.103183202	39390.0291	54293.20647	69196.38482	4064.389317	5602.146874	7139.904531
C11.05	Irapuato	68	0.154774803	36107.86789	50272.92621	64437.98553	5588.588122	7780.982226	9973.376486
C11.05	Irapuato	69	0.206366403	31608.07411	44293.77412	56979.47517	6522.844571	9140.746856	11758.64936
C11.05	Irapuato	70	0.206366403	28099.66509	38902.32928	49704.99455	5798.82682	8028.133776	10257.44095
C11.05	Irapuato	71	0.412732807	24587.75093	34481.15354	44374.55725	10148.17145	14231.50328	18314.83556
C11.05	Irapuato	72	0.103183202	21863.4504	28976.4732	36089.4959	2255.940812	2989.885278	3723.829734
C11.05	Irapuato	73	0.051591601	17782.90208	24246.95164	30711.00234	917.4483859	1250.939051	1584.429774
C11.05	Irapuato	74	0.206366403	13811.94966	18884.42031	23956.89213	2850.322375	3897.109899	4943.897664
C11.05	Irapuato	75	0.257958004	7935.115184	11930.12034	15925.12669	2046.926476	3077.470033	4108.013899
C11.05	Irapuato	76	0.361141206	3654.873946	6821.56493	9988.255913	1319.925584	2463.548185	3607.170785
C11.05	Irapuato	77	0.257958004	3654.873946	6821.56493	9988.255913	942.8039888	1759.677275	2576.550561
C11.05	Irapuato	78	0.309549605	3654.873946	6821.56493	9988.255913	1131.364787	2111.61273	3091.860673
C11.05	Irapuato	79	0.412732807	3654.873946	6821.56493	9988.255913	1508.486382	2815.48364	4122.480897
C11.05	Irapuato	80	0.361141206	3654.873946	6821.56493	9988.255913	1319.925584	2463.548185	3607.170785
C11.05	Irapuato	81	0.257958004	3654.873946	6821.56493	9988.255913	942.8039888	1759.677275	2576.550561
C11.05	Irapuato	82	0.515916008	3654.873946	6821.56493	9988.255913	1885.607978	3519.35455	5153.101122
C11.05	Irapuato	83	0.206366403	3654.873946	6821.56493	9988.255913	754.243191	1407.74182	2061.240449
C11.05	Irapuato	84	0.61909921	3654.873946	6821.56493	9988.255913	2262.729573	4223.225459	6183.721346
C11.05	Irapuato	85	0.773874013	3654.873946	6821.56493	9988.255913	2828.411966	5279.031824	7729.651682
C11.05	Irapuato	86	0.361141206	3654.873946	6821.56493	9988.255913	1319.925584	2463.548185	3607.170785

City ID	City name	Age	Avoidable deaths from respiratory causes mean estimate	Lower value, lost productivity	Center value, lost productivity	Upper value, lost productivity	Lost productivity by age, lower value	Lost productivity by age, center value	Lost productivity by age, upper value
C11.05	Irapuato	87	0.464324408	3654.873946	6821.56493	9988.255913	1697.04718	3167.419095	4637.791009
C11.05	Irapuato	88	0.309549605	3654.873946	6821.56493	9988.255913	1131.364787	2111.61273	3091.860673
C11.05	Irapuato	89	0.206366403	3654.873946	6821.56493	9988.255913	754.243191	1407.74182	2061.240449
C11.05	Irapuato	90	0.257958004	3654.873946	6821.56493	9988.255913	942.8039888	1759.677275	2576.550561
C11.05	Irapuato	91	0.257958004	3654.873946	6821.56493	9988.255913	942.8039888	1759.677275	2576.550561
C11.05	Irapuato	92	0.361141206	3654.873946	6821.56493	9988.255913	1319.925584	2463.548185	3607.170785
C11.05	Irapuato	93	0.361141206	3654.873946	6821.56493	9988.255913	1319.925584	2463.548185	3607.170785
C11.05	Irapuato	94	0.154774803	3654.873946	6821.56493	9988.255913	565.6823933	1055.806365	1545.930336
C11.05	Irapuato	95	0.154774803	3654.873946	6821.56493	9988.255913	565.6823933	1055.806365	1545.930336
C11.05	Irapuato	97	0.103183202	3654.873946	6821.56493	9988.255913	377.1215955	703.8709099	1030.620224
C11.05	Irapuato	98	0.103183202	3654.873946	6821.56493	9988.255913	377.1215955	703.8709099	1030.620224
C11.05	Irapuato	100	0.206366403	3654.873946	6821.56493	9988.255913	754.243191	1407.74182	2061.240449
C11.05	Irapuato	101	0.051591601	3654.873946	6821.56493	9988.255913	188.5607978	351.935455	515.3101122
C11.05	Irapuato	102	0.051591601	3654.873946	6821.56493	9988.255913	188.5607978	351.935455	515.3101122
C11.05	Irapuato	104	0.051591601	3654.873946	6821.56493	9988.255913	188.5607978	351.935455	515.3101122
C11.06	Salamanca	30	0.043514162	173556.3867	213150.4168	257080.3119	7552.160771	9275.061821	11186.6344
C11.06	Salamanca	31	0.043514162	172444.5127	211573.0974	255167.6219	7503.778505	9206.426089	11103.4053
C11.06	Salamanca	41	0.043514162	151036.141	188842.593	232650.8959	6572.211146	8217.327233	10123.60883
C11.06	Salamanca	45	0.043514162	136165.1594	174871.8634	220333.7048	5925.112839	7609.402639	9587.636582
C11.06	Salamanca	46	0.043514162	133802.6104	171944.7413	216885.3646	5822.308498	7482.031371	9437.584947
C11.06	Salamanca	48	0.043514162	126512.1799	161902.4834	204505.3087	5505.071525	7045.050931	8898.877184
C11.06	Salamanca	49	0.043514162	124199.3619	152973.2626	195979.9164	5404.431186	6656.503368	8527.901879

City ID	City name	Age	Avoidable deaths from respiratory causes mean estimate	Lower value, lost productivity	Center value, lost productivity	Upper value, lost productivity	Lost productivity by age, lower value	Lost productivity by age, center value	Lost productivity by age, upper value
C11.06	Salamanca	52	0.043514162	116644.9217	145932.806	177409.3755	5075.70605	6350.1438	7719.820351
C11.06	Salamanca	54	0.043514162	108132.5508	134926.1242	164041.6725	4705.297358	5871.197262	7138.135953
C11.06	Salamanca	56	0.043514162	98178.61828	125211.219	154707.2041	4272.160326	5448.461302	6731.954381
C11.06	Salamanca	60	0.043514162	79059.70015	102335.6919	128384.2444	3440.21662	4453.051902	5586.53284
C11.06	Salamanca	62	0.043514162	70203.73922	92014.35148	116766.3734	3054.856899	4003.92742	5080.990917
C11.06	Salamanca	65	0.087028325	58159.07016	75079.77522	95214.63799	5061.486431	6534.067042	8286.370413
C11.06	Salamanca	66	0.087028325	57129.10772	74015.21918	94211.91184	4971.850526	6441.420513	8199.104836
C11.06	Salamanca	67	0.043514162	57504.33018	70451.25432	86808.0771	2502.252754	3065.627312	3777.380752
C11.06	Salamanca	68	0.130542487	50712.83202	62724.20196	78247.76627	6620.179203	8188.173304	10214.65799
C11.06	Salamanca	69	0.043514162	43717.58891	58709.53316	69430.6461	1902.334257	2554.696152	3021.2164
C11.06	Salamanca	70	0.043514162	39255.15025	52425.46375	61196.81975	1708.154977	2281.250136	2662.928344
C11.06	Salamanca	71	0.174056649	34658.83844	45952.87225	52715.9786	6032.601277	7998.402956	9175.566585
C11.06	Salamanca	73	0.087028325	21643.00052	29505.35193	32560.84241	1883.554072	2567.801343	2833.715559
C11.06	Salamanca	74	0.130542487	15797.77646	22906.07551	25063.30655	2062.281025	2990.216059	3271.826363
C11.06	Salamanca	75	0.043514162	9777.195688	13559.02133	17340.84461	425.4464795	590.0094543	754.572326
C11.06	Salamanca	76	0.130542487	4854.288663	5698.92508	6543.560296	633.6909136	743.9518518	854.2126333
C11.06	Salamanca	77	0.130542487	4854.288663	5698.92508	6543.560296	633.6909136	743.9518518	854.2126333
C11.06	Salamanca	78	0.043514162	4854.288663	5698.92508	6543.560296	211.2303045	247.9839506	284.7375444
C11.06	Salamanca	79	0.043514162	4854.288663	5698.92508	6543.560296	211.2303045	247.9839506	284.7375444
C11.06	Salamanca	80	0.174056649	4854.288663	5698.92508	6543.560296	844.9212181	991.9358024	1138.950178
C11.06	Salamanca	81	0.304599136	4854.288663	5698.92508	6543.560296	1478.612132	1735.887654	1993.162811
C11.06	Salamanca	82	0.174056649	4854.288663	5698.92508	6543.560296	844.9212181	991.9358024	1138.950178

City ID	City name	Age	Avoidable deaths from respiratory causes mean estimate	Lower value, lost productivity	Center value, lost productivity	Upper value, lost productivity	Lost productivity by age, lower value	Lost productivity by age, center value	Lost productivity by age, upper value
C11.06	Salamanca	83	0.174056649	4854.288663	5698.92508	6543.560296	844.9212181	991.9358024	1138.950178
C11.06	Salamanca	84	0.174056649	4854.288663	5698.92508	6543.560296	844.9212181	991.9358024	1138.950178
C11.06	Salamanca	85	0.217570811	4854.288663	5698.92508	6543.560296	1056.151523	1239.919753	1423.687722
C11.06	Salamanca	86	0.261084974	4854.288663	5698.92508	6543.560296	1267.381827	1487.903704	1708.425267
C11.06	Salamanca	87	0.087028325	4854.288663	5698.92508	6543.560296	422.460609	495.9679012	569.4750888
C11.06	Salamanca	88	0.087028325	4854.288663	5698.92508	6543.560296	422.460609	495.9679012	569.4750888
C11.06	Salamanca	89	0.261084974	4854.288663	5698.92508	6543.560296	1267.381827	1487.903704	1708.425267
C11.06	Salamanca	90	0.174056649	4854.288663	5698.92508	6543.560296	844.9212181	991.9358024	1138.950178
C11.06	Salamanca	91	0.130542487	4854.288663	5698.92508	6543.560296	633.6909136	743.9518518	854.2126333
C11.06	Salamanca	92	0.043514162	4854.288663	5698.92508	6543.560296	211.2303045	247.9839506	284.7375444
C11.06	Salamanca	93	0.130542487	4854.288663	5698.92508	6543.560296	633.6909136	743.9518518	854.2126333
C11.06	Salamanca	94	0.043514162	4854.288663	5698.92508	6543.560296	211.2303045	247.9839506	284.7375444
C11.06	Salamanca	95	0.043514162	4854.288663	5698.92508	6543.560296	211.2303045	247.9839506	284.7375444
C11.06	Salamanca	96	0.130542487	4854.288663	5698.92508	6543.560296	633.6909136	743.9518518	854.2126333
C11.06	Salamanca	100	0.087028325	4854.288663	5698.92508	6543.560296	422.460609	495.9679012	569.4750888
C11.06	Salamanca	103	0.043514162	4854.288663	5698.92508	6543.560296	211.2303045	247.9839506	284.7375444
C11.06	Salamanca	109	0.043514162	4854.288663	5698.92508	6543.560296	211.2303045	247.9839506	284.7375444
M05.01	La Laguna	34	0.008047451	192327.0263	207316.0866	222305.1429	1547.742333	1668.366063	1788.98976
M05.01	La Laguna	35	0.004023726	190527.4149	205442.986	220358.5529	766.6300244	826.6461886	886.6623362
M05.01	La Laguna	37	0.008047451	186027.7307	200896.3928	215765.0531	1497.04906	1616.703891	1736.358707
M05.01	La Laguna	38	0.008047451	183437.49	198327.1024	213216.714	1476.204225	1596.027652	1715.851073
M05.01	La Laguna	39	0.004023726	180388.9343	195270.4262	210151.9185	725.8355612	785.7145999	845.5936406

City ID	City name	Age	Avoidable deaths from respiratory causes mean estimate	Lower value, lost productivity	Center value, lost productivity	Upper value, lost productivity	Lost productivity by age, lower value	Lost productivity by age, center value	Lost productivity by age, upper value
M05.01	La Laguna	41	0.008047451	182118.014	197218.8862	212319.759	1465.585806	1587.109336	1708.632871
M05.01	La Laguna	42	0.004023726	179263.1491	194372.7395	209482.3304	721.3057105	782.1025549	842.8994017
M05.01	La Laguna	43	0.008047451	176227.8905	191310.7737	206393.6573	1418.185326	1539.56409	1660.942858
M05.01	La Laguna	44	0.012071177	173684.9835	188752.4855	203819.9882	2096.58211	2278.464587	2460.347072
M05.01	La Laguna	45	0.004023726	170540.3624	185578.2359	200616.1112	686.2076107	746.7158864	807.2241695
M05.01	La Laguna	46	0.008047451	167278.4676	182287.1682	197295.8708	1346.165283	1466.947067	1587.728866
M05.01	La Laguna	47	0.016094902	163337.7198	178291.6192	193245.5194	2628.904615	2869.586163	3110.267723
M05.01	La Laguna	49	0.008047451	155402.9338	170084.972	184767.0121	1250.597506	1368.750489	1486.903489
M05.01	La Laguna	50	0.004023726	151649.8626	166171.7776	180693.6947	610.1974245	668.6296248	727.0618333
M05.01	La Laguna	51	0.012071177	146594.3244	161023.5299	175452.7376	1769.565979	1943.743467	2117.920981
M05.01	La Laguna	52	0.004023726	141978.2338	156188.4637	170398.6946	571.2814447	628.4595096	685.6375783
M05.01	La Laguna	53	0.012071177	137255.3103	151358.4263	165461.5433	1656.83309	1827.074294	1997.315511
M05.01	La Laguna	55	0.024142353	126810.7261	140574.2054	154337.6846	3061.50934	3393.792119	3726.074894
M05.01	La Laguna	56	0.016094902	122659.8639	136245.834	149831.8039	1974.198506	2192.863365	2411.52822
M05.01	La Laguna	57	0.020118628	117619.7329	131012.1883	144404.6434	2366.347614	2635.785436	2905.223254
M05.01	La Laguna	58	0.03621353	112652.6157	125821.7856	138990.9553	4079.548858	4556.450985	5033.353104
M05.01	La Laguna	59	0.032189804	107676.9295	120603.9423	133530.9562	3466.099286	3882.217299	4298.335344
M05.01	La Laguna	60	0.012071177	103236.8076	115804.1778	128371.5479	1246.189736	1397.892682	1549.595625
M05.01	La Laguna	61	0.028166079	97528.16596	109707.3407	121886.5152	2746.986002	3090.025595	3433.065183
M05.01	La Laguna	62	0.028166079	91499.93441	103209.1184	114918.3022	2577.194357	2906.996156	3236.797949
M05.01	La Laguna	63	0.024142353	85725.92467	96952.06342	108178.202	2069.625552	2340.650959	2611.676361
M05.01	La Laguna	64	0.024142353	80610.41556	91335.83774	102061.2597	1946.125125	2205.062055	2463.998981

City ID	City name	Age	Avoidable deaths from respiratory causes mean estimate	Lower value, lost productivity	Center value, lost productivity	Upper value, lost productivity	Lost productivity by age, lower value	Lost productivity by age, center value	Lost productivity by age, upper value
M05.01	La Laguna	65	0.032189804	74751.70411	84810.40944	94869.1158	2406.242725	2730.030481	3053.818269
M05.01	La Laguna	66	0.024142353	67869.5983	77357.11589	86844.63577	1638.531814	1867.582815	2096.633871
M05.01	La Laguna	67	0.016094902	61750.41059	70671.16561	79591.92174	993.8668154	1137.445494	1281.024191
M05.01	La Laguna	68	0.048284706	55117.92114	63500.52827	71883.13655	2661.35264	3066.104365	3470.856145
M05.01	La Laguna	69	0.032189804	49913.38946	57393.90033	64874.41363	1606.702237	1847.498418	2088.294677
M05.01	La Laguna	70	0.028166079	44086.31	50968.92896	57851.54917	1241.738479	1435.594866	1629.451289
M05.01	La Laguna	71	0.020118628	37632.00627	43972.27419	50312.54218	757.1043226	884.6618124	1012.219303
M05.01	La Laguna	72	0.044260981	30378.63203	35607.89796	40837.16395	1344.588052	1576.040491	1807.492933
M05.01	La Laguna	73	0.032189804	25060.45756	29469.38473	33878.31196	806.6912238	948.6137265	1090.536231
M05.01	La Laguna	74	0.056332157	19957.38146	23303.14691	26648.91244	1124.242355	1312.716542	1501.190732
M05.01	La Laguna	75	0.020118628	14187.90108	16296.38562	18404.869	285.4410992	327.8609147	370.2807067
M05.01	La Laguna	76	0.076450785	7582.502511	8859.07636	10135.64901	579.6882704	677.2833435	774.8783247
M05.01	La Laguna	77	0.044260981	7582.502511	8859.07636	10135.64901	335.6089987	392.1114094	448.6137669
M05.01	La Laguna	78	0.03621353	7582.502511	8859.07636	10135.64901	274.5891807	320.8184259	367.0476275
M05.01	La Laguna	79	0.044260981	7582.502511	8859.07636	10135.64901	335.6089987	392.1114094	448.6137669
M05.01	La Laguna	80	0.056332157	7582.502511	8859.07636	10135.64901	427.1387256	499.0508847	570.9629761
M05.01	La Laguna	81	0.084498236	7582.502511	8859.07636	10135.64901	640.7080884	748.576327	856.4444642
M05.01	La Laguna	82	0.056332157	7582.502511	8859.07636	10135.64901	427.1387256	499.0508847	570.9629761
M05.01	La Laguna	83	0.028166079	7582.502511	8859.07636	10135.64901	213.5693628	249.5254423	285.4814881
M05.01	La Laguna	84	0.060355883	7582.502511	8859.07636	10135.64901	457.6486345	534.6973764	611.7460458
M05.01	La Laguna	85	0.052308432	7582.502511	8859.07636	10135.64901	396.6288166	463.4043929	530.1799064
M05.01	La Laguna	86	0.03621353	7582.502511	8859.07636	10135.64901	274.5891807	320.8184259	367.0476275

City ID	City name	Age	Avoidable deaths from respiratory causes mean estimate	Lower value, lost productivity	Center value, lost productivity	Upper value, lost productivity	Lost productivity by age, lower value	Lost productivity by age, center value	Lost productivity by age, upper value
M05.01	La Laguna	87	0.068403334	7582.502511	8859.07636	10135.64901	518.6684525	605.9903599	693.3121853
M05.01	La Laguna	88	0.040237255	7582.502511	8859.07636	10135.64901	305.0990897	356.4649176	407.8306972
M05.01	La Laguna	89	0.052308432	7582.502511	8859.07636	10135.64901	396.6288166	463.4043929	530.1799064
M05.01	La Laguna	90	0.032189804	7582.502511	8859.07636	10135.64901	244.0792718	285.1719341	326.2645578
M05.01	La Laguna	91	0.060355883	7582.502511	8859.07636	10135.64901	457.6486345	534.6973764	611.7460458
M05.01	La Laguna	92	0.016094902	7582.502511	8859.07636	10135.64901	122.0396359	142.585967	163.1322789
M05.01	La Laguna	93	0.03621353	7582.502511	8859.07636	10135.64901	274.5891807	320.8184259	367.0476275
M05.01	La Laguna	94	0.024142353	7582.502511	8859.07636	10135.64901	183.0594538	213.8789506	244.6984183
M05.01	La Laguna	95	0.020118628	7582.502511	8859.07636	10135.64901	152.5495448	178.2324588	203.9153486
M05.01	La Laguna	96	0.016094902	7582.502511	8859.07636	10135.64901	122.0396359	142.585967	163.1322789
M05.01	La Laguna	97	0.016094902	7582.502511	8859.07636	10135.64901	122.0396359	142.585967	163.1322789
M05.01	La Laguna	98	0.016094902	7582.502511	8859.07636	10135.64901	122.0396359	142.585967	163.1322789
M05.01	La Laguna	101	0.004023726	7582.502511	8859.07636	10135.64901	30.50990897	35.64649176	40.78306972
M05.01	La Laguna	102	0.004023726	7582.502511	8859.07636	10135.64901	30.50990897	35.64649176	40.78306972
M05.01	La Laguna	106	0.004023726	7582.502511	8859.07636	10135.64901	30.50990897	35.64649176	40.78306972
M09.01	Valle de México	30	0.949964498	229822.1346	239150.5128	248478.8945	218322.8688	227184.4969	236046.1283
M09.01	Valle de México	31	0.76977445	227694.8983	237085.6781	246476.4616	175273.7151	182502.4975	189731.2827
M09.01	Valle de México	32	0.789187921	225532.9578	234987.101	244441.2468	177987.8862	185448.9818	192910.0795
M09.01	Valle de México	33	1.192235614	223181.7553	232701.5018	242221.2508	266085.237	277435.0178	288784.8016

City ID	City name	Age	Avoidable deaths from respiratory causes mean estimate	Lower value, lost productivity	Center value, lost productivity	Upper value, lost productivity	Lost productivity by age, lower value	Lost productivity by age, center value	Lost productivity by age, upper value
M09.01	Valle de México	34	0.913067784	220892.804	230458.4471	240024.093	201690.103	210424.1836	219158.2667
M09.01	Valle de México	35	1.737133229	218060.5472	227658.2556	237255.9656	378800.2225	395472.7208	412145.2217
M09.01	Valle de México	36	0.987711781	215431.2395	225090.7654	234750.2916	212783.9731	222324.8007	231865.6285
M09.01	Valle de México	37	1.177065208	212654.5371	222355.7129	232056.8902	250308.2569	261727.1734	273146.0917
M09.01	Valle de México	38	0.993695488	209884.1177	219644.3708	229404.6255	208560.9007	218259.6201	227958.3412
M09.01	Valle de México	39	1.544587718	206908.3439	216599.8468	226291.3502	319588.0866	334557.463	349526.8401
M09.01	Valle de México	40	1.484626986	212704.7298	222677.3246	232649.9186	315787.1819	330592.7653	345398.3476
M09.01	Valle de México	41	1.528044409	209857.7644	219896.9694	229936.1736	320671.9837	336012.3347	351352.6845
M09.01	Valle de México	42	2.385416313	206802.316	216929.2029	227056.089	493309.6182	517466.4595	541623.2988
M09.01	Valle de México	43	1.754659633	203397.7547	213630.0918	223862.4281	356893.8296	374848.0986	392802.366
M09.01	Valle de México	44	1.888654351	200051.6431	210361.81	220671.9748	377828.4063	397300.7478	416773.0853
M09.01	Valle de México	45	2.344174988	196108.1107	206228.3707	216348.6297	459711.7281	483435.3884	507159.0465
M09.01	Valle de México	46	1.566479979	192257.0764	202468.62	212680.1615	301166.8611	317163.0398	333159.215

City ID	City name	Age	Avoidable deaths from respiratory causes mean estimate	Lower value, lost productivity	Center value, lost productivity	Upper value, lost productivity	Lost productivity by age, lower value	Lost productivity by age, center value	Lost productivity by age, upper value
M09.01	Valle de México	47	1.096083472	187975.9846	198176.371	208376.7552	206037.3698	217217.8448	228398.3172
M09.01	Valle de México	48	2.098868246	183308.4664	193276.0139	203243.5578	384740.3193	405660.8882	426581.4496
M09.01	Valle de México	49	2.491401744	178987.6931	188637.6556	198287.6157	445930.2507	469972.1842	494014.1115
M09.01	Valle de México	50	3.532943445	174524.3331	184002.0308	193479.7259	616584.5985	650068.7684	683552.9292
M09.01	Valle de México	51	2.689779075	169951.4556	179461.9024	188972.3478	457131.8691	482712.8698	508293.8669
M09.01	Valle de México	52	3.143662049	165265.4176	174812.4457	184359.4723	519538.6214	549551.2512	579563.8766
M09.01	Valle de México	53	3.292622371	160459.3637	170008.4343	179557.5034	528332.0905	559773.574	591215.0528
M09.01	Valle de México	54	3.389880505	155606.9173	165142.3231	174677.7274	527488.8553	559812.7416	592136.6229
M09.01	Valle de México	55	4.232107495	150342.9818	159818.2835	169293.5848	636267.6602	676368.1553	716468.6492
M09.01	Valle de México	56	3.055126062	144920.829	154064.2334	163207.6364	442751.4015	470685.6548	498619.9034
M09.01	Valle de México	57	3.954099273	139188.0717	148240.1365	157292.1997	550363.4531	586156.216	621948.9726
M09.01	Valle de México	58	3.972368043	133220.8521	142208.973	151197.0922	529202.2556	564906.3796	600610.4971
M09.01	Valle de México	59	3.81764169	127214.1477	136003.7341	144793.3188	485658.0339	519213.5254	552769.0105

City ID	City name	Age	Avoidable deaths from respiratory causes mean estimate	Lower value, lost productivity	Center value, lost productivity	Upper value, lost productivity	Lost productivity by age, lower value	Lost productivity by age, center value	Lost productivity by age, upper value
M09.01	Valle de México	60	4.44709139	120666.2806	128923.3152	137180.3469	536613.9775	573333.7651	610053.5396
M09.01	Valle de México	61	4.92488432	114456.0587	122623.1191	130790.1765	563682.8487	603904.6766	644126.4893
M09.01	Valle de México	62	4.99324729	108274.8276	116358.2301	124441.6293	540642.9898	581005.4169	621367.8283
M09.01	Valle de México	63	6.30779255	101108.7367	108686.1368	116263.535	637772.9359	685569.6043	733366.26
M09.01	Valle de México	64	5.427823138	94373.35273	101651.6919	108930.0289	512241.8676	551747.4051	591252.9313
M09.01	Valle de México	65	6.859078675	88369.46809	95442.55795	102515.6457	606133.1341	654648.0139	703162.879
M09.01	Valle de México	66	6.458400764	82175.81947	88594.20999	95012.59953	530724.3753	572176.9135	613629.4454
M09.01	Valle de México	67	6.727083645	75057.55895	81248.50716	87439.45438	504918.4772	546565.5037	588212.5234
M09.01	Valle de México	68	8.864213885	68138.5239	74104.64248	80070.76003	603994.4496	656879.4008	709764.3428
M09.01	Valle de México	69	7.255455102	61194.44494	66859.96031	72525.47461	443993.5477	485099.4401	526205.3248
M09.01	Valle de México	70	8.811395314	53988.19233	59204.03396	64419.87326	475711.3049	521670.1474	567628.9693
M09.01	Valle de México	71	8.818347071	47009.1239	51074.25427	55139.38348	414542.77	450390.5006	486238.2208
M09.01	Valle de México	72	9.598279571	39725.32338	42975.32801	46225.33267	381294.7599	412489.2129	443683.6662

City ID	City name	Age	Avoidable deaths from respiratory causes mean estimate	Lower value, lost productivity	Center value, lost productivity	Upper value, lost productivity	Lost productivity by age, lower value	Lost productivity by age, center value	Lost productivity by age, upper value
M09.01	Valle de México	73	9.10493331	31786.64758	34385.24358	36983.83839	289415.3063	313075.3497	336735.3821
M09.01	Valle de México	74	10.77874797	23930.62114	25986.95698	28043.29159	257942.134	280106.8597	302271.5722
M09.01	Valle de México	75	11.42840056	16078.55021	17655.39909	19232.24794	183752.1122	201772.9728	219793.833
M09.01	Valle de México	76	10.88193657	7810.453078	8350.219961	8889.988046	84992.85499	90866.56398	96740.28604
M09.01	Valle de México	77	11.14157226	7810.453078	8350.219961	8889.988046	87020.72737	93034.57911	99048.44422
M09.01	Valle de México	78	13.65871072	7810.453078	8350.219961	8889.988046	106680.7192	114053.2389	121425.775
M09.01	Valle de México	79	14.46240181	7810.453078	8350.219961	8889.988046	112957.9107	120764.2363	128570.5792
M09.01	Valle de México	80	14.66444067	7810.453078	8350.219961	8889.988046	114535.9258	122451.3052	130366.7023
M09.01	Valle de México	81	13.83422453	7810.453078	8350.219961	8889.988046	108051.5616	115518.8178	122986.0907
M09.01	Valle de México	82	13.5557371	7810.453078	8350.219961	8889.988046	105876.4485	113193.3865	120510.3407
M09.01	Valle de México	83	13.86404455	7810.453078	8350.219961	8889.988046	108284.4694	115767.8215	123251.1903
M09.01	Valle de México	84	16.88723148	7810.453078	8350.219961	8889.988046	131896.9291	141012.0974	150127.286
M09.01	Valle de México	85	15.98271633	7810.453078	8350.219961	8889.988046	124832.2559	133459.1969	142086.1571

City ID	City name	Age	Avoidable deaths from respiratory causes mean estimate	Lower value, lost productivity	Center value, lost productivity	Upper value, lost productivity	Lost productivity by age, lower value	Lost productivity by age, center value	Lost productivity by age, upper value
M09.01	Valle de México	86	16.28870215	7810.453078	8350.219961	8889.988046	127222.1439	136014.2459	144806.3674
M09.01	Valle de México	87	15.5272218	7810.453078	8350.219961	8889.988046	121274.6373	129655.7174	138036.8162
M09.01	Valle de México	88	13.42619136	7810.453078	8350.219961	8889.988046	104864.6376	112111.6511	119358.6807
M09.01	Valle de México	89	16.1260227	7810.453078	8350.219961	8889.988046	125951.5436	134655.8367	143360.149
M09.01	Valle de México	90	11.96236118	7810.453078	8350.219961	8889.988046	93431.46071	99888.34712	106345.2479
M09.01	Valle de México	91	10.32492069	7810.453078	8350.219961	8889.988046	80642.30861	86215.35887	91788.42153
M09.01	Valle de México	92	11.95507608	7810.453078	8350.219961	8889.988046	93374.56074	99827.5149	106280.4834
M09.01	Valle de México	93	9.317402788	7810.453078	8350.219961	8889.988046	72773.13728	77802.36275	82831.5994
M09.01	Valle de México	94	7.978488718	7810.453078	8350.219961	8889.988046	62315.61176	66622.13575	70928.66933
M09.01	Valle de México	95	6.363843492	7810.453078	8350.219961	8889.988046	49704.50099	53139.49296	56574.49257
M09.01	Valle de México	96	5.041739234	7810.453078	8350.219961	8889.988046	39378.26772	42099.63159	44821.00152
M09.01	Valle de México	97	4.427307606	7810.453078	8350.219961	8889.988046	34579.27832	36968.99235	39358.71169
M09.01	Valle de México	98	2.866167785	7810.453078	8350.219961	8889.988046	22386.069	23933.13145	25480.19734

City ID	City name	Age	Avoidable deaths from respiratory causes mean estimate	Lower value, lost productivity	Center value, lost productivity	Upper value, lost productivity	Lost productivity by age, lower value	Lost productivity by age, center value	Lost productivity by age, upper value
M09.01	Valle de México	99	1.422763819	7810.453078	8350.219961	8889.988046	11112.43005	11880.39084	12648.35335
M09.01	Valle de México	100	2.240616623	7810.453078	8350.219961	8889.988046	17500.231	18709.64165	19919.055
M09.01	Valle de México	101	1.464799923	7810.453078	8350.219961	8889.988046	11440.75107	12231.40156	13022.0538
M09.01	Valle de México	102	0.595690259	7810.453078	8350.219961	8889.988046	4652.610816	4974.14469	5295.67928
M09.01	Valle de México	103	0.646552713	7810.453078	8350.219961	8889.988046	5049.869625	5398.857367	5747.845886
M09.01	Valle de México	104	0.330522543	7810.453078	8350.219961	8889.988046	2581.530812	2759.935935	2938.341454
M09.01	Valle de México	105	0.365337181	7810.453078	8350.219961	8889.988046	2853.448909	3050.645821	3247.843171
M09.01	Valle de México	106	0.101759815	7810.453078	8350.219961	8889.988046	794.7902619	849.7168403	904.6435408
M09.01	Valle de México	107	0.103876524	7810.453078	8350.219961	8889.988046	811.3227171	867.3918247	923.4610571
M09.01	Valle de México	109	0.050536163	7810.453078	8350.219961	8889.988046	394.7103333	421.9880807	449.2658889
M09.01	Valle de México	110	0.06974367	7810.453078	8350.219961	8889.988046	544.72966	582.3749833	620.0203903
M09.01	Valle de México	111	0.050536163	NA	NA	NA	NA	NA	NA
M09.01	Valle de México	112	0.052777566	NA	NA	NA	NA	NA	NA

City ID	City name	Age	Avoidable deaths from respiratory causes mean estimate	Lower value, lost productivity	Center value, lost productivity	Upper value, lost productivity	Lost productivity by age, lower value	Lost productivity by age, center value	Lost productivity by age, upper value
M09.01	Valle de México	120	0.06974367	NA	NA	NA	NA	NA	NA
M11.01	Celaya	33	0.050668157	164562.8828	199044.3331	233525.7809	8338.098037	10085.20959	11832.32101
M11.01	Celaya	34	0.050668157	162869.4688	197819.3323	232769.1945	8252.29587	10023.14105	11793.98616
M11.01	Celaya	37	0.050668157	156289.953	192358.0974	228426.2404	7918.923926	9746.430343	11573.93669
M11.01	Celaya	38	0.050668157	153573.0801	189835.5421	226098.0026	7781.264982	9618.61711	11455.96916
M11.01	Celaya	45	0.050668157	137721.0302	172823.9839	207926.9401	6978.070824	8756.672807	10535.27491
M11.01	Celaya	46	0.050668157	133451.1703	168798.2523	204145.338	6761.724892	8552.696403	10343.6681
M11.01	Celaya	48	0.152004472	124782.9167	160266.5509	195750.1878	18967.56136	24361.23244	29754.90394
M11.01	Celaya	49	0.050668157	120325.9872	155593.3074	190860.6292	6096.696048	7883.626176	9670.556385
M11.01	Celaya	50	0.152004472	116212.2612	151335.8685	186459.4762	17664.7834	23003.72878	28342.67422
M11.01	Celaya	53	0.202672629	103114.1522	138576.4078	174038.664	20898.41634	28085.64494	35272.87362
M11.01	Celaya	55	0.202672629	96478.33716	131086.7458	165695.1536	19553.51826	26567.69543	33581.87244
M11.01	Celaya	56	0.101336315	92083.46136	126430.1725	160776.8829	9331.398613	12811.96774	16292.53679
M11.01	Celaya	57	0.050668157	86469.13353	120485.5583	154501.9822	4381.231661	6104.781222	7828.330741
M11.01	Celaya	58	0.101336315	82689.51903	116702.5967	150715.6735	8379.451118	11826.21106	15272.97091
M11.01	Celaya	59	0.050668157	77179.29911	110760.4652	144341.6292	3910.532869	5612.028677	7313.524379
M11.01	Celaya	60	0.202672629	74977.53985	108473.3182	141969.0943	15195.89514	21984.5726	28773.24962
M11.01	Celaya	61	0.152004472	71578.76051	104834.3428	138089.9229	10880.2917	15935.28893	20990.28582
M11.01	Celaya	62	0.101336315	67197.55749	99633.80659	132070.0546	6809.552829	10096.52277	13383.49261
M11.01	Celaya	65	0.152004472	53197.89069	83664.90807	114131.9217	8086.317284	12717.44017	17348.5625
M11.01	Celaya	66	0.152004472	49370.67305	78448.66083	107526.6435	7504.563087	11924.54727	16344.53067

City ID	City name	Age	Avoidable deaths from respiratory causes mean estimate	Lower value, lost productivity	Center value, lost productivity	Upper value, lost productivity	Lost productivity by age, lower value	Lost productivity by age, center value	Lost productivity by age, upper value
M11.01	Celaya	67	0.050668157	47340.72625	67026.01698	86711.2988	2398.667365	3396.084773	4393.501729
M11.01	Celaya	68	0.202672629	45873.57588	63166.44084	80459.29785	9297.318239	12802.10865	16306.89745
M11.01	Celaya	69	0.101336315	38931.50529	54497.89436	70064.27773	3945.17527	5522.615771	7100.055693
M11.01	Celaya	70	0.304008944	31236.64862	43814.21007	56391.77058	9496.220558	13319.91173	17143.60262
M11.01	Celaya	71	0.152004472	28060.88471	37982.89267	47904.89843	4265.379964	5773.569544	7281.758791
M11.01	Celaya	72	0.050668157	21223.51218	29731.8699	38240.22535	1075.356254	1506.459061	1937.561754
M11.01	Celaya	73	0.253340787	16775.86407	23859.38141	30942.89766	4250.010599	6044.554455	7839.098032
M11.01	Celaya	74	0.253340787	12504.25299	17181.44276	21858.63139	3167.837289	4352.760223	5537.682871
M11.01	Celaya	75	0.152004472	7886.116283	11881.30584	15876.49423	1198.724941	1806.01162	2413.298122
M11.01	Celaya	76	0.152004472	4092.690627	5208.45214	6324.212453	622.1072776	791.7080173	961.3085745
M11.01	Celaya	77	0.304008944	4092.690627	5208.45214	6324.212453	1244.214555	1583.416035	1922.617149
M11.01	Celaya	78	0.253340787	4092.690627	5208.45214	6324.212453	1036.845463	1319.513362	1602.180957
M11.01	Celaya	79	0.202672629	4092.690627	5208.45214	6324.212453	829.4763702	1055.61069	1281.744766
M11.01	Celaya	80	0.202672629	4092.690627	5208.45214	6324.212453	829.4763702	1055.61069	1281.744766
M11.01	Celaya	81	0.202672629	4092.690627	5208.45214	6324.212453	829.4763702	1055.61069	1281.744766
M11.01	Celaya	82	0.405345259	4092.690627	5208.45214	6324.212453	1658.95274	2111.22138	2563.489532
M11.01	Celaya	83	0.354677101	4092.690627	5208.45214	6324.212453	1451.583648	1847.318707	2243.05334
M11.01	Celaya	84	0.354677101	4092.690627	5208.45214	6324.212453	1451.583648	1847.318707	2243.05334
M11.01	Celaya	85	0.304008944	4092.690627	5208.45214	6324.212453	1244.214555	1583.416035	1922.617149
M11.01	Celaya	86	0.304008944	4092.690627	5208.45214	6324.212453	1244.214555	1583.416035	1922.617149
M11.01	Celaya	87	0.253340787	4092.690627	5208.45214	6324.212453	1036.845463	1319.513362	1602.180957
M11.01	Celaya	88	0.354677101	4092.690627	5208.45214	6324.212453	1451.583648	1847.318707	2243.05334

City ID	City name	Age	Avoidable deaths from respiratory causes mean estimate	Lower value, lost productivity	Center value, lost productivity	Upper value, lost productivity	Lost productivity by age, lower value	Lost productivity by age, center value	Lost productivity by age, upper value
M11.01	Celaya	89	0.253340787	4092.690627	5208.45214	6324.212453	1036.845463	1319.513362	1602.180957
M11.01	Celaya	90	0.253340787	4092.690627	5208.45214	6324.212453	1036.845463	1319.513362	1602.180957
M11.01	Celaya	91	0.253340787	4092.690627	5208.45214	6324.212453	1036.845463	1319.513362	1602.180957
M11.01	Celaya	92	0.202672629	4092.690627	5208.45214	6324.212453	829.4763702	1055.61069	1281.744766
M11.01	Celaya	93	0.050668157	4092.690627	5208.45214	6324.212453	207.3690925	263.9026724	320.4361915
M11.01	Celaya	94	0.202672629	4092.690627	5208.45214	6324.212453	829.4763702	1055.61069	1281.744766
M11.01	Celaya	97	0.101336315	4092.690627	5208.45214	6324.212453	414.7381851	527.8053449	640.872383
M11.01	Celaya	98	0.152004472	4092.690627	5208.45214	6324.212453	622.1072776	791.7080173	961.3085745
M11.01	Celaya	99	0.050668157	4092.690627	5208.45214	6324.212453	207.3690925	263.9026724	320.4361915
M11.01	Celaya	100	0.101336315	4092.690627	5208.45214	6324.212453	414.7381851	527.8053449	640.872383
M11.03	León	30	0.042849035	187781.5834	198982.5843	210183.5812	8046.259547	8526.211619	9006.163524
M11.03	León	31	0.042849035	186458.1199	197673.63	208889.136	7989.550415	8470.124193	8950.697797
M11.03	León	32	0.042849035	184694.6863	195894.2826	207093.8748	7913.988988	8393.880876	8873.772587
M11.03	León	34	0.085698069	180684.343	191903.351	203122.3559	15484.2993	16445.74662	17407.19367
M11.03	León	35	0.130009774	178717.998	189969.7573	201221.5145	23235.08662	24697.9253	26160.76372
M11.03	León	36	0.128547104	176162.2265	187420.061	198677.8947	22645.14397	24092.30599	25539.4679
M11.03	León	37	0.085698069	173646.3712	184897.7224	196149.0729	14881.1587	15845.37778	16809.59678
M11.03	León	38	0.042849035	170775.9669	182056.19	193336.4134	7317.585298	7800.931966	8284.27865
M11.03	León	39	0.085698069	167850.3829	179092.6634	190334.9444	14384.45369	15347.89543	16311.3372
M11.03	León	40	0.08716074	172965.4184	184550.7333	196136.0486	15075.79386	16085.57848	17095.36313
M11.03	León	43	0.085698069	163531.7818	175251.9438	186972.1076	14014.35792	15018.75317	16023.14858
M11.03	León	44	0.128547104	160232.5947	171992.4708	183752.3488	20597.43593	22109.13395	23620.8322

City ID	City name	Age	Avoidable deaths from respiratory causes mean estimate	Lower value, lost productivity	Center value, lost productivity	Upper value, lost productivity	Lost productivity by age, lower value	Lost productivity by age, center value	Lost productivity by age, upper value
M11.03	León	45	0.128547104	156957.1016	168764.4325	180571.7652	20176.38079	21694.17898	23211.9774
M11.03	León	46	0.085698069	152881.0446	164736.3658	176591.6888	13101.61031	14117.58844	15133.56674
M11.03	León	47	0.042849035	148998.8142	160865.0424	172731.2713	6384.455332	6892.911752	7401.368204
M11.03	León	48	0.302868583	145540.2031	157244.8872	168949.5708	44079.55514	47624.53624	51169.51719
M11.03	León	49	0.128547104	141798.004	153384.0464	164970.087	18227.7227	19717.07489	21206.42685
M11.03	León	50	0.128547104	137170.028	148420.6028	159671.177	17632.80979	19079.03859	20525.26732
M11.03	León	51	0.042849035	133308.9592	144519.8906	155730.8214	5712.160192	6192.537779	6672.915342
M11.03	León	52	0.172858809	129396.7395	140440.1265	151483.5117	22367.36627	24276.313	26185.25941
M11.03	León	53	0.128547104	125481.5954	136352.4227	147223.2483	16130.29563	17527.709	18925.12213
M11.03	León	54	0.172858809	120675.2546	131214.7818	141754.3083	20859.78079	22681.63091	24503.48091
M11.03	León	55	0.301405913	116411.9433	126848.1075	137284.2723	35087.24799	38232.7696	41378.29137
M11.03	León	56	0.644198189	112493.08	122788.7571	133084.436	72467.83837	79100.29492	85732.75261
M11.03	León	57	0.171396138	107401.8155	117502.8703	127603.9269	18408.25639	20139.53817	21870.82027
M11.03	León	58	0.171396138	102410.419	112318.6454	122226.8737	17552.75031	19250.98205	20949.21411
M11.03	León	59	0.26148222	97507.52666	107178.8444	116850.1654	25496.48453	28025.36217	30554.24064
M11.03	León	60	0.385641311	92290.1904	101285.0976	110280.0093	35590.90998	39059.71777	42528.5273
M11.03	León	61	0.387103982	87333.51671	96248.23836	105162.9646	33807.15204	37258.07628	40709.00232
M11.03	León	62	0.514188414	81728.17643	90364.84608	99001.51927	42023.68142	46464.55689	50905.43418
M11.03	León	63	0.387103982	76095.09324	84285.83525	92476.58214	29456.71357	32627.38241	35798.05314
M11.03	León	64	0.299943242	71413.20693	79228.67022	87044.13855	21419.90877	23764.10417	26108.30107
M11.03	León	65	0.472802051	66392.89601	73838.50612	81284.12019	31390.69738	34910.9971	38431.2987
M11.03	León	66	0.47133938	61572.35978	68885.17562	76197.99552	29021.47786	32468.29594	35915.11593

City ID	City name	Age	Avoidable deaths from respiratory causes mean estimate	Lower value, lost productivity	Center value, lost productivity	Upper value, lost productivity	Lost productivity by age, lower value	Lost productivity by age, center value	Lost productivity by age, upper value
M11.03	León	67	0.387103982	55897.6949	62956.42097	70015.14998	21638.22026	24370.68122	27103.14332
M11.03	León	68	0.214245173	49666.94307	55483.68985	61300.43967	10640.90279	11887.1127	13133.32327
M11.03	León	69	0.557037449	44286.31316	49395.98393	54505.65783	24669.13489	27515.41286	30361.69257
M11.03	León	70	0.472802051	39086.37259	43117.83488	47149.30163	18480.11711	20386.20074	22292.28649
M11.03	León	71	0.729896258	34344.34761	37911.51018	41478.67735	25067.81079	27671.4694	30275.13137
M11.03	León	72	0.688509894	29159.43271	32203.86341	35248.2976	20076.55793	22172.67858	24268.80165
M11.03	León	73	0.858443361	22098.14974	24465.85195	26833.55653	18970.00993	21002.54818	23035.08846
M11.03	León	74	0.55850012	16179.19411	18052.63793	19926.08295	9036.081846	10082.40044	11128.71971
M11.03	León	75	0.815594327	11034.15262	12222.94043	13411.72823	8999.392274	9968.960865	10938.52946
M11.03	León	76	0.64566086	5687.562856	6308.283484	6929.004112	3672.236722	4073.011736	4473.786751
M11.03	León	77	0.815594327	5687.562856	6308.283484	6929.004112	4638.743997	5145.00022	5651.256443
M11.03	León	78	0.687047223	5687.562856	6308.283484	6929.004112	3907.624266	4334.08865	4760.553034
M11.03	León	79	0.691435236	5687.562856	6308.283484	6929.004112	3932.581365	4361.769479	4790.957593
M11.03	León	80	0.901292396	5687.562856	6308.283484	6929.004112	5126.157151	5685.607933	6245.058715
M11.03	León	81	1.029839499	5687.562856	6308.283484	6929.004112	5857.276883	6496.519503	7135.762124
M11.03	León	82	0.818519669	5687.562856	6308.283484	6929.004112	4655.382063	5163.454106	5671.526149
M11.03	León	83	1.029839499	5687.562856	6308.283484	6929.004112	5857.276883	6496.519503	7135.762124
M11.03	León	84	1.288396377	5687.562856	6308.283484	6929.004112	7327.835378	8127.569587	8927.303795
M11.03	León	85	1.119925581	5687.562856	6308.283484	6929.004112	6369.647136	7064.808046	7759.968956
M11.03	León	86	0.814131656	5687.562856	6308.283484	6929.004112	4630.424964	5135.773277	5641.12159
M11.03	León	87	0.772745292	5687.562856	6308.283484	6929.004112	4395.03742	4874.696363	5354.355306
M11.03	León	88	0.775670634	5687.562856	6308.283484	6929.004112	4411.675486	4893.15025	5374.625013

City ID	City name	Age	Avoidable deaths from respiratory causes mean estimate	Lower value, lost productivity	Center value, lost productivity	Upper value, lost productivity	Lost productivity by age, lower value	Lost productivity by age, center value	Lost productivity by age, upper value
M11.03	León	89	0.858443361	5687.562856	6308.283484	6929.004112	4882.450574	5415.304077	5948.157579
M11.03	León	90	0.991378478	5687.562856	6308.283484	6929.004112	5638.527405	6253.896476	6869.265547
M11.03	León	91	1.204160979	5687.562856	6308.283484	6929.004112	6848.741257	7596.188816	8343.636376
M11.03	León	92	0.899829725	5687.562856	6308.283484	6929.004112	5117.838118	5676.38099	6234.923862
M11.03	León	93	0.515651085	5687.562856	6308.283484	6929.004112	2932.797958	3252.873223	3572.948489
M11.03	León	94	0.605737167	5687.562856	6308.283484	6929.004112	3445.168211	3821.161766	4197.155321
M11.03	León	95	0.55850012	5687.562856	6308.283484	6929.004112	3176.504535	3523.17708	3869.849625
M11.03	León	96	0.344254947	5687.562856	6308.283484	6929.004112	1957.97165	2171.657797	2385.343944
M11.03	León	97	0.215707843	5687.562856	6308.283484	6929.004112	1226.851918	1360.746227	1494.640535
M11.03	León	98	0.085698069	5687.562856	6308.283484	6929.004112	487.4131541	540.6077134	593.8022726
M11.03	León	99	0.171396138	5687.562856	6308.283484	6929.004112	974.8263082	1081.215427	1187.604545
M11.03	León	101	0.128547104	5687.562856	6308.283484	6929.004112	731.1197312	810.91157	890.7034089
M11.03	León	102	0.085698069	5687.562856	6308.283484	6929.004112	487.4131541	540.6077134	593.8022726
M11.03	León	104	0.042849035	5687.562856	6308.283484	6929.004112	243.7065771	270.3038567	296.9011363
M13.01	Pachuca	30	0.041384125	231489.1722	246345.1764	261201.182	9579.976822	10194.77956	10809.58235
M13.01	Pachuca	38	0.041384125	210596.2818	225593.445	240590.6085	8715.342838	9335.987312	9956.631802
M13.01	Pachuca	40	0.041384125	213447.1799	229021.8647	244596.5513	8833.324759	9477.869462	10122.41424
M13.01	Pachuca	41	0.041384125	210240.5544	225686.6364	241132.7214	8700.621371	9339.843957	9979.066668
M13.01	Pachuca	42	0.041384125	207142.4614	222387.8812	237633.304	8572.4095	9203.327857	9834.246342
M13.01	Pachuca	44	0.041384125	199808.661	214939.9059	230071.154	8268.906591	8895.099917	9521.293379
M13.01	Pachuca	45	0.041384125	195797.9978	210906.976	226015.9576	8102.928803	8728.200644	9353.472624
M13.01	Pachuca	47	0.08276825	187382.9875	202662.1191	217941.2531	15509.36193	16773.98891	18038.61609

City ID	City name	Age	Avoidable deaths from respiratory causes mean estimate	Lower value, lost productivity	Center value, lost productivity	Upper value, lost productivity	Lost productivity by age, lower value	Lost productivity by age, center value	Lost productivity by age, upper value
M13.01	Pachuca	48	0.041384125	182583.8433	197514.0174	212444.1927	7556.07258	8173.944772	8791.81701
M13.01	Pachuca	50	0.08276825	173966.2803	188774.8744	203583.4697	14398.88456	15624.56597	16850.24749
M13.01	Pachuca	51	0.124152375	168742.7661	183437.4949	198132.2237	20949.81514	22774.20062	24598.58609
M13.01	Pachuca	53	0.041384125	156997.7738	171527.8377	186057.9016	6497.215486	7098.529465	7699.843445
M13.01	Pachuca	54	0.124152375	150737.1343	165250.9591	179764.7838	18714.37319	20516.29901	22318.22482
M13.01	Pachuca	56	0.041384125	138323.2649	152805.1538	167287.0428	5724.387275	6323.707576	6923.027877
M13.01	Pachuca	57	0.08276825	131895.4838	146263.3141	160631.1445	10916.75836	12105.95853	13295.1587
M13.01	Pachuca	58	0.041384125	125982.6033	139706.1063	153429.6107	5213.679793	5781.614959	6349.550176
M13.01	Pachuca	59	0.041384125	119681.3121	133252.2611	146823.2114	4952.906372	5514.528221	6076.150122
M13.01	Pachuca	60	0.124152375	113221.7241	126525.1918	139828.662	14056.74593	15708.40303	17360.06045
M13.01	Pachuca	62	0.124152375	102340.4084	114816.2812	127292.1542	12705.80474	14254.71398	15803.62324
M13.01	Pachuca	63	0.041384125	95209.51066	107234.8354	119260.1604	3940.162284	4437.819825	4935.477376
M13.01	Pachuca	65	0.1655365	81560.30805	93266.0675	104971.8272	13501.20791	15438.93836	17376.66884
M13.01	Pachuca	66	0.041384125	75979.95226	86579.96225	97179.97123	3144.363837	3583.035974	4021.70807
M13.01	Pachuca	67	0.041384125	70210.64602	79972.97053	89735.294	2905.606147	3309.611404	3713.616618
M13.01	Pachuca	68	0.124152375	62518.74006	71051.13981	79583.53849	7761.850047	8821.16774	9880.485299
M13.01	Pachuca	69	0.041384125	56074.72817	64090.79364	72106.858	2320.603556	2652.341411	2984.07922
M13.01	Pachuca	71	0.1655365	44094.09145	49835.04917	55576.00696	7299.181557	8249.519603	9199.857661
M13.01	Pachuca	72	0.24830475	36647.17555	41718.09569	46789.01589	9099.667749	10358.8013	11617.93487
M13.01	Pachuca	73	0.041384125	29174.56911	33585.39366	37996.21828	1207.364013	1389.902127	1572.440244
M13.01	Pachuca	74	0.289688875	20408.99619	23954.75693	27500.51649	5912.259138	6939.426574	7966.593672
M13.01	Pachuca	75	0.1655365	12295.16605	14499.63272	16704.09819	2035.298751	2400.218448	2765.137945

City ID	City name	Age	Avoidable deaths from respiratory causes mean estimate	Lower value, lost productivity	Center value, lost productivity	Upper value, lost productivity	Lost productivity by age, lower value	Lost productivity by age, center value	Lost productivity by age, upper value
M13.01	Pachuca	76	0.041384125	7535.546577	8299.691568	9063.836558	311.852001	343.4754727	375.0989445
M13.01	Pachuca	77	0.24830475	7535.546577	8299.691568	9063.836558	1871.112006	2060.852836	2250.593667
M13.01	Pachuca	78	0.24830475	7535.546577	8299.691568	9063.836558	1871.112006	2060.852836	2250.593667
M13.01	Pachuca	79	0.24830475	7535.546577	8299.691568	9063.836558	1871.112006	2060.852836	2250.593667
M13.01	Pachuca	80	0.24830475	7535.546577	8299.691568	9063.836558	1871.112006	2060.852836	2250.593667
M13.01	Pachuca	81	0.496609499	7535.546577	8299.691568	9063.836558	3742.224012	4121.705673	4501.187334
M13.01	Pachuca	83	0.041384125	7535.546577	8299.691568	9063.836558	311.852001	343.4754727	375.0989445
M13.01	Pachuca	84	0.24830475	7535.546577	8299.691568	9063.836558	1871.112006	2060.852836	2250.593667
M13.01	Pachuca	85	0.1655365	7535.546577	8299.691568	9063.836558	1247.408004	1373.901891	1500.395778
M13.01	Pachuca	86	0.041384125	7535.546577	8299.691568	9063.836558	311.852001	343.4754727	375.0989445
M13.01	Pachuca	87	0.041384125	7535.546577	8299.691568	9063.836558	311.852001	343.4754727	375.0989445
M13.01	Pachuca	88	0.124152375	7535.546577	8299.691568	9063.836558	935.556003	1030.426418	1125.296833
M13.01	Pachuca	89	0.124152375	7535.546577	8299.691568	9063.836558	935.556003	1030.426418	1125.296833
M13.01	Pachuca	90	0.124152375	7535.546577	8299.691568	9063.836558	935.556003	1030.426418	1125.296833
M13.01	Pachuca	91	0.289688875	7535.546577	8299.691568	9063.836558	2182.964007	2404.328309	2625.692611
M13.01	Pachuca	92	0.1655365	7535.546577	8299.691568	9063.836558	1247.408004	1373.901891	1500.395778
M13.01	Pachuca	93	0.24830475	7535.546577	8299.691568	9063.836558	1871.112006	2060.852836	2250.593667
M13.01	Pachuca	94	0.124152375	7535.546577	8299.691568	9063.836558	935.556003	1030.426418	1125.296833
M13.01	Pachuca	95	0.1655365	7535.546577	8299.691568	9063.836558	1247.408004	1373.901891	1500.395778
M13.01	Pachuca	96	0.041384125	7535.546577	8299.691568	9063.836558	311.852001	343.4754727	375.0989445
M13.01	Pachuca	97	0.08276825	7535.546577	8299.691568	9063.836558	623.704002	686.9509455	750.1978889
M13.01	Pachuca	98	0.08276825	7535.546577	8299.691568	9063.836558	623.704002	686.9509455	750.1978889

City ID	City name	Age	Avoidable deaths from respiratory causes mean estimate	Lower value, lost productivity	Center value, lost productivity	Upper value, lost productivity	Lost productivity by age, lower value	Lost productivity by age, center value	Lost productivity by age, upper value
M13.01	Pachuca	100	0.124152375	7535.546577	8299.691568	9063.836558	935.556003	1030.426418	1125.296833
M13.01	Pachuca	101	0.041384125	7535.546577	8299.691568	9063.836558	311.852001	343.4754727	375.0989445
M13.01	Pachuca	107	0.041384125	7535.546577	8299.691568	9063.836558	311.852001	343.4754727	375.0989445
M13.02	Tula	41	NA	NA	NA	NA	NA	NA	NA
M13.02	Tula	51	NA	NA	NA	NA	NA	NA	NA
M13.02	Tula	58	NA	NA	NA	NA	NA	NA	NA
M13.02	Tula	60	NA	NA	NA	NA	NA	NA	NA
M13.02	Tula	64	NA	NA	NA	NA	NA	NA	NA
M13.02	Tula	66	NA	NA	NA	NA	NA	NA	NA
M13.02	Tula	70	NA	NA	NA	NA	NA	NA	NA
M13.02	Tula	71	NA	NA	NA	NA	NA	NA	NA
M13.02	Tula	77	NA	NA	NA	NA	NA	NA	NA
M13.02	Tula	83	NA	NA	NA	NA	NA	NA	NA
M13.02	Tula	84	NA	NA	NA	NA	NA	NA	NA
M13.02	Tula	85	NA	NA	NA	NA	NA	NA	NA
M13.02	Tula	88	NA	NA	NA	NA	NA	NA	NA
M13.02	Tula	89	NA	NA	NA	NA	NA	NA	NA
M13.02	Tula	90	NA	NA	NA	NA	NA	NA	NA
M14.01	Guadalajara	30	0.062039726	232071.5909	241182.4004	250293.2059	14397.65785	14962.88998	15528.12185
M14.01	Guadalajara	31	0.040306987	230160.9258	239303.3578	248445.7856	9277.093411	9645.597298	10014.10101
M14.01	Guadalajara	32	0.018730317	227745.1831	236575.7144	245406.2413	4265.739487	4431.138137	4596.536706
M14.01	Guadalajara	33	0.044536466	225296.19	233803.795	242311.3955	10033.89606	10412.79472	10791.69317

City ID	City name	Age	Avoidable deaths from respiratory causes mean estimate	Lower value, lost productivity	Center value, lost productivity	Upper value, lost productivity	Lost productivity by age, lower value	Lost productivity by age, center value	Lost productivity by age, upper value
M14.01	Guadalajara	34	0.049158183	222694.3101	231214.8192	239735.325	10947.24771	11366.10046	11784.95304
M14.01	Guadalajara	35	0.126533566	220161.5628	228702.1004	237242.6332	27857.82756	28938.49224	30019.15631
M14.01	Guadalajara	36	0.134229058	217612.7091	226161.7462	234710.7783	29209.94896	30357.47815	31505.00668
M14.01	Guadalajara	37	0.119050814	214268.0616	222699.8525	231131.6397	25508.78706	26512.59863	27516.40975
M14.01	Guadalajara	38	0.114836016	211304.7443	219716.5479	228128.3477	24265.3951	25231.37312	26197.3507
M14.01	Guadalajara	39	0.058645065	208348.0458	216738.7913	225129.5328	12218.58476	12710.66058	13202.73616
M14.01	Guadalajara	40	0.209329824	214350.5662	223036.6194	231722.6696	44869.9664	46688.21638	48506.46577
M14.01	Guadalajara	41	0.125170074	211061.7145	219713.8067	228365.8958	26418.61044	27501.59346	28584.5761
M14.01	Guadalajara	42	0.14308655	207239.5021	215894.8232	224550.1425	29653.18545	30891.64549	32130.10527
M14.01	Guadalajara	43	0.170457682	203683.0158	212376.4925	221069.966	34719.33479	36201.20467	37683.07403
M14.01	Guadalajara	44	0.092060672	199734.6379	208434.5195	217134.3979	18387.7049	19188.62185	19989.53849
M14.01	Guadalajara	45	0.106439757	195463.4635	203987.7985	212512.1289	20805.08357	21712.41171	22619.73937
M14.01	Guadalajara	46	0.309920807	191153.0278	199635.5422	208118.0506	59242.30064	61871.20834	64500.1142
M14.01	Guadalajara	47	0.06449384	186839.0174	195292.6692	203746.315	12049.96568	12595.17415	13140.38222
M14.01	Guadalajara	48	0.237006238	182722.6746	191113.0286	199503.3776	43306.4138	45294.98004	47283.54508
M14.01	Guadalajara	49	0.250476583	178080.6446	186408.6565	194736.6644	44605.03129	46691.00325	48776.97421
M14.01	Guadalajara	50	0.202138536	173446.8148	181692.4338	189938.0488	35060.28523	36727.04259	38393.79913
M14.01	Guadalajara	51	0.289822046	168578.0411	176738.2352	184898.4251	48857.63272	51222.63686	53587.63979
M14.01	Guadalajara	52	0.325648702	163534.1234	171633.5233	179732.9188	53254.67505	55892.2341	58529.79173
M14.01	Guadalajara	53	0.279351553	158470.7452	166462.9879	174455.2262	44269.04885	46501.69428	48734.33845
M14.01	Guadalajara	54	0.463922788	152769.6835	160652.8729	168536.0577	70873.33749	74530.52869	78187.71776
M14.01	Guadalajara	55	0.369185533	147245.619	155025.7289	162805.8353	54360.95231	57233.25634	60105.55907

City ID	City name	Age	Avoidable deaths from respiratory causes mean estimate	Lower value, lost productivity	Center value, lost productivity	Upper value, lost productivity	Lost productivity by age, lower value	Lost productivity by age, center value	Lost productivity by age, upper value
M14.01	Guadalajara	56	0.228172107	141614.0276	149302.7559	156991.4794	32312.37105	34066.72439	35821.07663
M14.01	Guadalajara	57	0.526012913	135918.1645	143491.486	151064.8026	71494.7097	75478.37463	79462.03693
M14.01	Guadalajara	58	0.436047452	129789.2859	137147.0798	144504.8685	56594.28746	59802.63471	63010.97973
M14.01	Guadalajara	59	0.336526115	123579.0241	130765.1704	137951.3127	41587.56881	44005.89472	46424.21927
M14.01	Guadalajara	60	0.627179546	117057.7586	124031.4731	131005.1847	73416.23187	77790.00296	82163.77221
M14.01	Guadalajara	61	0.565999015	111346.7571	118128.1243	124909.4884	63022.1548	66860.40194	70698.64737
M14.01	Guadalajara	62	0.593214289	105477.4878	112005.2027	118532.9144	62570.75291	66443.08661	70315.41846
M14.01	Guadalajara	63	0.530177311	99151.21565	105492.0231	111832.8273	52567.72489	55929.47713	59291.22767
M14.01	Guadalajara	64	0.810449776	92435.56824	98533.24418	104630.9181	74914.38557	79856.24568	84798.1041
M14.01	Guadalajara	65	0.881972682	85856.3259	91668.38178	97480.43552	75722.93401	80849.00852	85975.08115
M14.01	Guadalajara	66	0.703211071	79472.51555	84982.89609	90493.27444	55885.95277	59760.91338	63635.87244
M14.01	Guadalajara	67	0.770298858	73072.47255	78265.54499	83458.61515	56287.64217	60287.85994	64288.07596
M14.01	Guadalajara	68	0.746512957	66496.37624	71253.69797	76011.0186	49640.40647	53191.80878	56743.21028
M14.01	Guadalajara	69	1.031518802	59490.90552	63804.16591	68117.42516	61365.98759	65815.19677	70264.40479
M14.01	Guadalajara	70	0.940608018	51966.9483	55850.58044	59734.21143	48880.52825	52533.50379	56186.47823
M14.01	Guadalajara	71	0.884912405	44681.41085	48098.65592	51515.89978	39539.13475	42563.0973	45587.05879
M14.01	Guadalajara	72	0.975460794	38131.79439	41072.61745	44013.43927	37196.07042	40064.72802	42933.38442
M14.01	Guadalajara	73	0.975109025	31213.35888	33706.94138	36200.52384	30436.42795	32867.94275	35299.45751
M14.01	Guadalajara	74	1.309369414	24015.07386	26007.33213	27999.59036	31444.60319	34053.20522	36661.80721
M14.01	Guadalajara	75	1.164567711	16839.98804	18211.68872	19583.39061	19611.30633	21208.74465	22806.18438
M14.01	Guadalajara	76	1.037826334	8292.801223	9006.638771	9720.47632	8606.487488	9347.326894	10088.1663
M14.01	Guadalajara	77	1.330425762	8292.801223	9006.638771	9720.47632	11032.95639	11982.66425	12932.37211

City ID	City name	Age	Avoidable deaths from respiratory causes mean estimate	Lower value, lost productivity	Center value, lost productivity	Upper value, lost productivity	Lost productivity by age, lower value	Lost productivity by age, center value	Lost productivity by age, upper value
M14.01	Guadalajara	78	1.628103071	8292.801223	9006.638771	9720.47632	13501.53514	14663.73624	15825.93735
M14.01	Guadalajara	79	1.550520102	8292.801223	9006.638771	9720.47632	12858.155	13964.97446	15071.79393
M14.01	Guadalajara	80	1.532735014	8292.801223	9006.638771	9720.47632	12710.6668	13804.79061	14898.91441
M14.01	Guadalajara	81	1.259372406	8292.801223	9006.638771	9720.47632	10443.72503	11342.71234	12241.69965
M14.01	Guadalajara	82	1.193477057	8292.801223	9006.638771	9720.47632	9897.267994	10749.21673	11601.16547
M14.01	Guadalajara	83	1.527144461	8292.801223	9006.638771	9720.47632	12664.30546	13754.43852	14844.57157
M14.01	Guadalajara	84	1.539004376	8292.801223	9006.638771	9720.47632	12762.65737	13861.25648	14959.85559
M14.01	Guadalajara	85	1.943865449	8292.801223	9006.638771	9720.47632	16120.08977	17507.69392	18895.29807
M14.01	Guadalajara	86	1.640623351	8292.801223	9006.638771	9720.47632	13605.36333	14776.50188	15947.64043
M14.01	Guadalajara	87	1.446193905	8292.801223	9006.638771	9720.47632	11992.99858	13025.34609	14057.69361
M14.01	Guadalajara	88	1.435980325	8292.801223	9006.638771	9720.47632	11908.2994	12933.35607	13958.41275
M14.01	Guadalajara	89	1.62744398	8292.801223	9006.638771	9720.47632	13496.06943	14657.80005	15819.53067
M14.01	Guadalajara	90	1.857306829	8292.801223	9006.638771	9720.47632	15402.27634	16728.09169	18053.90705
M14.01	Guadalajara	91	1.566131021	8292.801223	9006.638771	9720.47632	12987.61324	14105.57637	15223.5395
M14.01	Guadalajara	92	1.489714779	8292.801223	9006.638771	9720.47632	12353.90854	13417.32289	14480.73723
M14.01	Guadalajara	93	1.26043474	8292.801223	9006.638771	9720.47632	10452.53475	11352.2804	12252.02604
M14.01	Guadalajara	94	1.32354667	8292.801223	9006.638771	9720.47632	10975.90944	11920.70675	12865.50406
M14.01	Guadalajara	95	0.742423522	8292.801223	9006.638771	9720.47632	6156.770694	6686.740481	7216.710269
M14.01	Guadalajara	96	0.64779328	8292.801223	9006.638771	9720.47632	5372.020904	5834.440071	6296.859237
M14.01	Guadalajara	97	0.718727266	8292.801223	9006.638771	9720.47632	5960.262349	6473.316859	6986.371368
M14.01	Guadalajara	98	0.321575292	8292.801223	9006.638771	9720.47632	2666.759977	2896.312496	3125.865014
M14.01	Guadalajara	99	0.280861385	8292.801223	9006.638771	9720.47632	2329.127638	2529.61704	2730.106443

City ID	City name	Age	Avoidable deaths from respiratory causes mean estimate	Lower value, lost productivity	Center value, lost productivity	Upper value, lost productivity	Lost productivity by age, lower value	Lost productivity by age, center value	Lost productivity by age, upper value
M14.01	Guadalajara	100	0.220185151	8292.801223	9006.638771	9720.47632	1825.951689	1983.128117	2140.304545
M14.01	Guadalajara	101	0.2318827	8292.801223	9006.638771	9720.47632	1922.957139	2088.483717	2254.010295
M14.01	Guadalajara	102	0.136960994	8292.801223	9006.638771	9720.47632	1135.790298	1233.558198	1331.326098
M14.01	Guadalajara	103	0.162630708	8292.801223	9006.638771	9720.47632	1348.664136	1464.756042	1580.847948
M14.01	Guadalajara	104	0.193809717	8292.801223	9006.638771	9720.47632	1607.225456	1745.574109	1883.922763
M14.01	Guadalajara	105	0.024579092	8292.801223	9006.638771	9720.47632	203.8295212	221.3749997	238.9204782
M15.02	Toluca	30	0.026069682	191361.5579	203668.4963	215975.4352	4988.73493	5309.572897	5630.410878
M15.02	Toluca	32	0.021727082	188072.3845	200454.3669	212836.3512	4086.264038	4355.288379	4624.312761
M15.02	Toluca	33	0.020638409	185932.5314	198381.6002	210830.6696	3837.351704	4094.280681	4351.209672
M15.02	Toluca	36	0.021727082	179892.5736	192437.6707	204982.7673	3908.540618	4181.108966	4453.677303
M15.02	Toluca	38	0.020093619	175381.5316	187978.9591	200576.3886	3524.049688	3777.177598	4030.305548
M15.02	Toluca	39	0.021727082	172625.3277	185268.5735	197911.82	3750.644574	4025.345406	4300.046256
M15.02	Toluca	40	0.086908326	177499.818	190542.8181	203585.8191	15426.21209	16559.7574	17693.30279
M15.02	Toluca	41	0.042365491	174781.3334	187857.1568	200932.9809	7404.697	7958.660675	8512.624387
M15.02	Toluca	42	0.021727082	171445.343	184550.174	197655.0058	3725.00695	4009.736681	4294.466432
M15.02	Toluca	43	0.020093619	168184.9231	181220.9547	194256.9884	3379.443778	3641.384831	3903.325927
M15.02	Toluca	44	0.041820701	165062.4827	178085.0706	191107.6606	6903.028676	7447.642423	7992.256263
M15.02	Toluca	45	0.063547782	161721.0229	174647.4636	187573.9065	10277.01234	11098.45898	11919.90575
M15.02	Toluca	46	0.089617464	157908.8699	170743.4774	183578.0872	14151.39247	15301.59744	16451.80263
M15.02	Toluca	47	0.043454163	154141.1084	166872.5353	179603.9647	6698.072866	7251.30637	7804.539979
M15.02	Toluca	48	0.06259526	150420.657	163132.0202	175843.386	9415.620173	10211.29127	11006.96252
M15.02	Toluca	49	0.021727082	146303.9009	158889.9746	171476.0495	3178.756788	3452.215436	3725.674114

City ID	City name	Age	Avoidable deaths from respiratory causes mean estimate	Lower value, lost productivity	Center value, lost productivity	Upper value, lost productivity	Lost productivity by age, lower value	Lost productivity by age, center value	Lost productivity by age, upper value
M15.02	Toluca	50	0.125735311	141928.4209	154499.2972	167070.1748	17845.41412	19426.01716	21006.62037
M15.02	Toluca	51	0.110800664	136725.7594	149286.8253	161847.8926	15149.30489	16541.07933	17932.85393
M15.02	Toluca	52	0.02077456	131818.3279	144243.5792	156668.8307	2738.467716	2996.59684	3254.725968
M15.02	Toluca	53	0.083641401	127224.6642	139477.1497	151729.6342	10641.24919	11666.06425	12690.87922
M15.02	Toluca	54	0.089345883	122469.6917	134491.6065	146513.5216	10942.1627	12016.2713	13090.37991
M15.02	Toluca	55	0.112978008	117230.2214	129131.7537	141033.2873	13244.4369	14589.04831	15933.65987
M15.02	Toluca	56	0.167963743	112324.079	124105.4414	135886.8054	18866.37274	20845.21448	22824.05647
M15.02	Toluca	57	0.043454163	108080.2952	119727.0541	131373.8159	4696.538776	5202.638941	5708.739226
M15.02	Toluca	58	0.200009488	102513.4826	113925.7576	125338.0354	20503.66914	22786.23241	25068.79626
M15.02	Toluca	59	0.158733577	97274.2777	108422.1982	119570.1204	15440.69409	17210.24339	18979.79296
M15.02	Toluca	60	0.129409967	92196.8869	103064.9688	113933.0524	11931.19613	13337.63426	14744.07261
M15.02	Toluca	61	0.204896878	86657.77098	97253.5003	107849.2302	17755.90675	19926.93861	22097.97059
M15.02	Toluca	62	0.116368086	80893.29451	91112.44925	101331.6033	9413.397887	10602.58137	11791.76477
M15.02	Toluca	63	0.085274864	75517.89432	85284.70387	95051.51149	6439.778149	7272.641503	8105.504692
M15.02	Toluca	64	0.132119105	70027.24672	79277.30982	88527.37095	9251.937178	10474.04724	11696.15704
M15.02	Toluca	65	0.227576482	64254.13141	73052.40659	81850.68097	14622.72916	16625.00968	18627.29001
M15.02	Toluca	66	0.108635408	58896.57394	67421.74895	75946.92314	6398.253328	7324.389192	8250.524966
M15.02	Toluca	67	0.296964896	53553.08905	61495.30894	69437.52921	15903.3875	18261.948	20620.50862
M15.02	Toluca	68	0.285692992	48165.62056	55290.98969	62416.35797	13760.58023	15796.24825	17831.91603
M15.02	Toluca	69	0.245097113	42475.3649	48945.97536	55416.58496	10410.58932	11996.51727	13582.445
M15.02	Toluca	70	0.206938072	37343.93999	43148.1997	48952.45728	7727.882957	8929.005272	10130.12715
M15.02	Toluca	71	0.284196588	32315.26607	37594.89915	42874.5288	9183.888349	10684.34206	12184.79479

City ID	City name	Age	Avoidable deaths from respiratory causes mean estimate	Lower value, lost productivity	Center value, lost productivity	Upper value, lost productivity	Lost productivity by age, lower value	Lost productivity by age, center value	Lost productivity by age, upper value
M15.02	Toluca	72	0.419036919	26261.34827	30637.23385	35013.11589	11004.47447	12838.13208	14671.78821
M15.02	Toluca	73	0.204228935	20825.03311	24331.01328	27836.99105	4253.074326	4969.09692	5685.119024
M15.02	Toluca	74	0.305799607	15515.69744	18162.2809	20808.86312	4744.694185	5554.018369	6363.342175
M15.02	Toluca	75	0.242387976	10392.6395	12177.34664	13962.05374	2519.050848	2951.642399	3384.233941
M15.02	Toluca	76	0.215761415	4541.873134	5574.77519	6607.678447	979.960974	1202.821383	1425.682051
M15.02	Toluca	77	0.453792885	4541.873134	5574.77519	6607.678447	2061.069712	2529.793316	2998.517465
M15.02	Toluca	78	0.318282618	4541.873134	5574.77519	6607.678447	1445.599271	1774.354042	2103.109195
M15.02	Toluca	79	0.510696486	4541.873134	5574.77519	6607.678447	2319.518648	2847.018098	3374.518162
M15.02	Toluca	80	0.325335439	4541.873134	5574.77519	6607.678447	1477.632291	1813.671936	2149.711971
M15.02	Toluca	81	0.393796606	4541.873134	5574.77519	6607.678447	1788.574224	2195.327548	2602.081345
M15.02	Toluca	82	0.440504697	4541.873134	5574.77519	6607.678447	2000.716449	2455.714657	2910.713393
M15.02	Toluca	83	0.376127546	4541.873134	5574.77519	6607.678447	1708.323598	2096.826514	2485.329882
M15.02	Toluca	84	0.407628501	4541.873134	5574.77519	6607.678447	1851.396936	2272.437252	2693.478058
M15.02	Toluca	85	0.3400097	4541.873134	5574.77519	6607.678447	1544.280919	1895.477637	2246.674763
M15.02	Toluca	86	0.341767224	4541.873134	5574.77519	6607.678447	1552.263372	1905.27544	2258.287919
M15.02	Toluca	87	0.356033927	4541.873134	5574.77519	6607.678447	1617.060929	1984.809105	2352.557708
M15.02	Toluca	88	0.250515389	4541.873134	5574.77519	6607.678447	1137.809114	1396.566975	1655.325136
M15.02	Toluca	89	0.506490036	4541.873134	5574.77519	6607.678447	2300.413486	2823.568085	3346.723293
M15.02	Toluca	90	0.330100396	4541.873134	5574.77519	6607.678447	1499.274119	1840.235496	2181.19727
M15.02	Toluca	91	0.463307454	4541.873134	5574.77519	6607.678447	2104.283678	2582.8349	3061.386678
M15.02	Toluca	92	0.397186684	4541.873134	5574.77519	6607.678447	1803.97153	2214.226473	2624.481893
M15.02	Toluca	93	0.248351041	4541.873134	5574.77519	6607.678447	1127.978923	1384.501224	1641.023824

City ID	City name	Age	Avoidable deaths from respiratory causes mean estimate	Lower value, lost productivity	Center value, lost productivity	Upper value, lost productivity	Lost productivity by age, lower value	Lost productivity by age, center value	Lost productivity by age, upper value
M15.02	Toluca	94	0.202595472	4541.873134	5574.77519	6607.678447	920.1629318	1129.424211	1338.685735
M15.02	Toluca	95	0.233008838	4541.873134	5574.77519	6607.678447	1058.296579	1298.971887	1539.647474
M15.02	Toluca	96	0.021727082	4541.873134	5574.77519	6607.678447	98.68164801	121.1235952	143.5655685
M15.02	Toluca	97	0.111889336	4541.873134	5574.77519	6607.678447	508.1871687	623.7578939	739.3287534
M15.02	Toluca	98	0.112978008	4541.873134	5574.77519	6607.678447	513.1317796	629.8269964	746.522349
M15.02	Toluca	99	0.043454163	4541.873134	5574.77519	6607.678447	197.363296	242.2471905	287.1311371
M15.02	Toluca	100	0.046708091	4541.873134	5574.77519	6607.678447	212.1422247	260.3871082	308.6320477
M15.02	Toluca	101	0.021727082	4541.873134	5574.77519	6607.678447	98.68164801	121.1235952	143.5655685
M15.02	Toluca	102	0.043454163	4541.873134	5574.77519	6607.678447	197.363296	242.2471905	287.1311371
M16.02	Morelia	32	0.049700555	225179.1627	238395.5244	251611.8882	11191.52941	11848.38993	12505.25055
M16.02	Morelia	34	0.074550833	221494.0868	234435.4614	247376.8395	16512.56864	17477.3589	18442.14941
M16.02	Morelia	37	0.024850278	213433.8173	226274.6296	239115.4444	5303.889614	5622.987363	5942.085177
M16.02	Morelia	40	0.074550833	212585.4553	225809.1369	239032.824	15848.42274	16834.25922	17820.09611
M16.02	Morelia	41	0.049700555	209458.4407	222744.2236	236030.0108	10410.2008	11070.51159	11730.82259
M16.02	Morelia	42	0.024850278	205949.7472	219265.9509	232582.1591	5117.908392	5448.819753	5779.731224
M16.02	Morelia	43	0.049700555	202483.9553	215819.3516	229154.7537	10063.565	10726.3416	11389.1185
M16.02	Morelia	46	0.024850278	191437.5176	204430.0997	217422.6882	4757.275459	5080.144731	5403.014161
M16.02	Morelia	47	0.024850278	187188.336	200097.3363	213006.3432	4651.682115	4972.474357	5293.266763
M16.02	Morelia	48	0.024850278	182256.4648	195134.8215	208013.1838	4529.123748	4849.154487	5169.185364
M16.02	Morelia	50	0.049700555	173473.6899	186173.0477	198872.4114	8621.738709	9252.903842	9884.069267
M16.02	Morelia	51	0.049700555	168458.3578	181084.8485	193711.3452	8372.473919	9000.017514	9627.56141
M16.02	Morelia	52	0.049700555	163243.1832	175728.5926	188214.007	8113.276845	8733.808623	9354.34065

City ID	City name	Age	Avoidable deaths from respiratory causes mean estimate	Lower value, lost productivity	Center value, lost productivity	Upper value, lost productivity	Lost productivity by age, lower value	Lost productivity by age, center value	Lost productivity by age, upper value
M16.02	Morelia	56	0.024850278	139901.6304	151835.7704	163769.9148	3476.594355	3773.161047	4069.727847
M16.02	Morelia	58	0.09940111	126477.3376	138138.4396	149799.5449	12571.9878	13731.11429	14890.24111
M16.02	Morelia	59	0.049700555	119482.332	130937.7647	142393.201	5938.338239	6507.679609	7077.021152
M16.02	Morelia	61	0.024850278	106174.8689	117031.7286	127888.592	2638.474968	2908.270946	3178.067015
M16.02	Morelia	62	0.09940111	99782.96202	110274.9573	120766.9577	9918.53723	10961.45322	12004.3697
M16.02	Morelia	63	0.049700555	94244.94225	104330.1849	114415.4316	4684.025958	5185.268119	5686.510477
M16.02	Morelia	64	0.074550833	87524.16524	97246.77524	106969.3893	6524.999413	7249.828086	7974.657063
M16.02	Morelia	65	0.024850278	80577.68218	89856.16988	99134.66177	2002.377772	2232.950767	2463.523867
M16.02	Morelia	66	0.124251388	74324.84428	83179.98972	92035.13949	9234.965072	10335.22918	11435.49383
M16.02	Morelia	67	0.049700555	67558.97836	75982.89634	84406.81877	3357.718736	3776.392136	4195.065758
M16.02	Morelia	69	0.09940111	54911.41923	62470.43984	70029.46515	5458.256049	6209.631092	6961.006602
M16.02	Morelia	70	0.124251388	46781.28093	53779.91996	60778.56258	5812.639092	6682.229706	7551.820766
M16.02	Morelia	71	0.173951943	39714.59321	45810.72123	51906.85171	6908.430667	7968.863983	9029.297727
M16.02	Morelia	72	0.09940111	32384.68682	36848.71653	41312.74754	3219.073832	3662.803342	4106.532982
M16.02	Morelia	73	0.09940111	25105.8824	28778.89798	32451.91367	2495.55259	2860.654418	3225.756256
M16.02	Morelia	74	0.09940111	19092.81096	21864.24882	24635.68555	1897.846611	2173.330612	2448.814501
M16.02	Morelia	75	0.124251388	13019.65346	14976.87135	16934.08806	1617.710015	1860.897054	2104.083948
M16.02	Morelia	76	0.149101666	6350.198347	7419.918956	8489.638365	946.825151	1106.322276	1265.819221
M16.02	Morelia	77	0.149101666	6350.198347	7419.918956	8489.638365	946.825151	1106.322276	1265.819221
M16.02	Morelia	78	0.223652499	6350.198347	7419.918956	8489.638365	1420.237727	1659.483414	1898.728832
M16.02	Morelia	79	0.223652499	6350.198347	7419.918956	8489.638365	1420.237727	1659.483414	1898.728832
M16.02	Morelia	80	0.298203331	6350.198347	7419.918956	8489.638365	1893.650302	2212.644552	2531.638443

City ID	City name	Age	Avoidable deaths from respiratory causes mean estimate	Lower value, lost productivity	Center value, lost productivity	Upper value, lost productivity	Lost productivity by age, lower value	Lost productivity by age, center value	Lost productivity by age, upper value
M16.02	Morelia	81	0.198802221	6350.198347	7419.918956	8489.638365	1262.433535	1475.096368	1687.758962
M16.02	Morelia	82	0.198802221	6350.198347	7419.918956	8489.638365	1262.433535	1475.096368	1687.758962
M16.02	Morelia	83	0.372754164	6350.198347	7419.918956	8489.638365	2367.062878	2765.805689	3164.548054
M16.02	Morelia	84	0.273353054	6350.198347	7419.918956	8489.638365	1735.84611	2028.257506	2320.668573
M16.02	Morelia	85	0.347903887	6350.198347	7419.918956	8489.638365	2209.258686	2581.418643	2953.578183
M16.02	Morelia	86	0.198802221	6350.198347	7419.918956	8489.638365	1262.433535	1475.096368	1687.758962
M16.02	Morelia	87	0.273353054	6350.198347	7419.918956	8489.638365	1735.84611	2028.257506	2320.668573
M16.02	Morelia	88	0.323053609	6350.198347	7419.918956	8489.638365	2051.454494	2397.031598	2742.608313
M16.02	Morelia	89	0.323053609	6350.198347	7419.918956	8489.638365	2051.454494	2397.031598	2742.608313
M16.02	Morelia	90	0.223652499	6350.198347	7419.918956	8489.638365	1420.237727	1659.483414	1898.728832
M16.02	Morelia	91	0.198802221	6350.198347	7419.918956	8489.638365	1262.433535	1475.096368	1687.758962
M16.02	Morelia	92	0.273353054	6350.198347	7419.918956	8489.638365	1735.84611	2028.257506	2320.668573
M16.02	Morelia	93	0.273353054	6350.198347	7419.918956	8489.638365	1735.84611	2028.257506	2320.668573
M16.02	Morelia	94	0.173951943	6350.198347	7419.918956	8489.638365	1104.629343	1290.709322	1476.789092
M16.02	Morelia	95	0.124251388	6350.198347	7419.918956	8489.638365	789.0209592	921.9352298	1054.849351
M16.02	Morelia	96	0.074550833	6350.198347	7419.918956	8489.638365	473.4125755	553.1611379	632.9096107
M16.02	Morelia	98	0.124251388	6350.198347	7419.918956	8489.638365	789.0209592	921.9352298	1054.849351
M16.02	Morelia	99	0.024850278	6350.198347	7419.918956	8489.638365	157.8041918	184.387046	210.9698702
M16.02	Morelia	101	0.024850278	6350.198347	7419.918956	8489.638365	157.8041918	184.387046	210.9698702
M16.02	Morelia	102	0.024850278	6350.198347	7419.918956	8489.638365	157.8041918	184.387046	210.9698702
M16.02	Morelia	103	0.024850278	6350.198347	7419.918956	8489.638365	157.8041918	184.387046	210.9698702
M16.02	Morelia	105	0.024850278	6350.198347	7419.918956	8489.638365	157.8041918	184.387046	210.9698702

City ID	City name	Age	Avoidable deaths from respiratory causes mean estimate	Lower value, lost productivity	Center value, lost productivity	Upper value, lost productivity	Lost productivity by age, lower value	Lost productivity by age, center value	Lost productivity by age, upper value
M16.02	Morelia	107	0.024850278	6350.198347	7419.918956	8489.638365	157.8041918	184.387046	210.9698702
M18.01	Tepic	35	0.017365118	210837.8558	222212.7391	233587.6207	3661.224314	3858.750508	4056.276673
M18.01	Tepic	39	0.017365118	198372.5738	209784.2925	221196.0106	3444.763217	3642.929062	3841.094897
M18.01	Tepic	44	0.017365118	191132.6184	202956.5469	214780.472	3319.040534	3524.364451	3729.68831
M18.01	Tepic	45	0.017365118	187393.1107	199239.6846	211086.2539	3254.103541	3459.820699	3665.537775
M18.01	Tepic	48	0.017365118	175305.7916	187094.4149	198883.0305	3044.205813	3248.916652	3453.627357
M18.01	Tepic	50	0.017365118	166303.9193	178035.8838	189767.8414	2887.887236	3091.614188	3295.34102
M18.01	Tepic	51	0.017365118	161007.0269	172712.7619	184418.4898	2795.906073	2999.177547	3202.448897
M18.01	Tepic	54	0.017365118	144735.3516	156265.3783	167795.3997	2513.346506	2713.566783	2913.78697
M18.01	Tepic	55	0.034730237	138435.0751	149830.2136	161225.3479	4807.882918	5203.638774	5599.394487
M18.01	Tepic	56	0.034730237	132657.6504	143949.3937	155241.1328	4607.231591	4999.39651	5391.56128
M18.01	Tepic	57	0.017365118	126745.301	137993.3967	149241.4881	2200.947149	2396.271662	2591.596099
M18.01	Tepic	59	0.017365118	114264.2274	125102.184	135940.1371	1984.21183	2172.414228	2360.616566
M18.01	Tepic	60	0.052095355	108757.7128	119396.8776	130036.039	5665.771652	6220.022722	6774.273609
M18.01	Tepic	61	0.034730237	102777.4898	113249.5465	123721.5983	3569.486541	3933.18355	4296.880389
M18.01	Tepic	63	0.017365118	90103.18651	100019.8916	109936.5928	1564.652495	1736.857252	1909.061941
M18.01	Tepic	65	0.069460473	78009.41068	87175.0406	96340.66763	5418.570587	6055.21958	6691.868372
M18.01	Tepic	66	0.052095355	70762.74171	79593.64439	88424.54408	3686.410148	4146.459158	4606.508012
M18.01	Tepic	67	0.017365118	63773.34544	72238.80259	80704.25791	1107.431689	1254.435355	1401.438988
M18.01	Tepic	68	0.017365118	57508.14101	65556.19136	73604.23982	998.6356733	1138.39102	1278.146334
M18.01	Tepic	69	0.086825592	51670.26718	59228.89594	66787.52153	4486.301517	5142.583931	5798.86607
M18.01	Tepic	70	0.034730237	45920.52348	53019.00944	60117.49213	1594.830647	1841.362745	2087.894728

City ID	City name	Age	Avoidable deaths from respiratory causes mean estimate	Lower value, lost productivity	Center value, lost productivity	Upper value, lost productivity	Lost productivity by age, lower value	Lost productivity by age, center value	Lost productivity by age, upper value
M18.01	Tepic	71	0.069460473	38791.88831	44571.78363	50351.67558	2694.502922	3095.977186	3497.451217
M18.01	Tepic	72	0.052095355	32253.53073	35470.9453	38688.35764	1680.259132	1847.871487	2015.483725
M18.01	Tepic	73	0.034730237	25920.12106	28441.42909	30962.73604	900.2119385	987.7775628	1075.34315
M18.01	Tepic	74	0.052095355	18621.82523	20557.01478	22492.202	970.1105957	1070.924982	1171.739247
M18.01	Tepic	75	0.069460473	11837.02678	13187.51359	14537.99923	822.2054827	916.0109356	1009.816307
M18.01	Tepic	76	0.069460473	5939.134888	6617.724565	7296.313041	412.5351203	459.6702804	506.8053572
M18.01	Tepic	77	0.156286065	5939.134888	6617.724565	7296.313041	928.2040207	1034.258131	1140.312054
M18.01	Tepic	78	0.069460473	5939.134888	6617.724565	7296.313041	412.5351203	459.6702804	506.8053572
M18.01	Tepic	79	0.086825592	5939.134888	6617.724565	7296.313041	515.6689004	574.5878506	633.5066965
M18.01	Tepic	80	0.10419071	5939.134888	6617.724565	7296.313041	618.8026805	689.5054207	760.2080357
M18.01	Tepic	81	0.156286065	5939.134888	6617.724565	7296.313041	928.2040207	1034.258131	1140.312054
M18.01	Tepic	82	0.069460473	5939.134888	6617.724565	7296.313041	412.5351203	459.6702804	506.8053572
M18.01	Tepic	83	0.173651183	5939.134888	6617.724565	7296.313041	1031.337801	1149.175701	1267.013393
M18.01	Tepic	84	0.121555828	5939.134888	6617.724565	7296.313041	721.9364605	804.4229908	886.909375
M18.01	Tepic	85	0.121555828	5939.134888	6617.724565	7296.313041	721.9364605	804.4229908	886.909375
M18.01	Tepic	86	0.086825592	5939.134888	6617.724565	7296.313041	515.6689004	574.5878506	633.5066965
M18.01	Tepic	87	0.156286065	5939.134888	6617.724565	7296.313041	928.2040207	1034.258131	1140.312054
M18.01	Tepic	88	0.086825592	5939.134888	6617.724565	7296.313041	515.6689004	574.5878506	633.5066965
M18.01	Tepic	89	0.10419071	5939.134888	6617.724565	7296.313041	618.8026805	689.5054207	760.2080357
M18.01	Tepic	90	0.052095355	5939.134888	6617.724565	7296.313041	309.4013402	344.7527103	380.1040179
M18.01	Tepic	91	0.191016302	5939.134888	6617.724565	7296.313041	1134.471581	1264.093271	1393.714732
M18.01	Tepic	92	0.086825592	5939.134888	6617.724565	7296.313041	515.6689004	574.5878506	633.5066965

City ID	City name	Age	Avoidable deaths from respiratory causes mean estimate	Lower value, lost productivity	Center value, lost productivity	Upper value, lost productivity	Lost productivity by age, lower value	Lost productivity by age, center value	Lost productivity by age, upper value
M18.01	Tepic	93	0.086825592	5939.134888	6617.724565	7296.313041	515.6689004	574.5878506	633.5066965
M18.01	Tepic	94	0.034730237	5939.134888	6617.724565	7296.313041	206.2675602	229.8351402	253.4026786
M18.01	Tepic	95	0.069460473	5939.134888	6617.724565	7296.313041	412.5351203	459.6702804	506.8053572
M18.01	Tepic	96	0.086825592	5939.134888	6617.724565	7296.313041	515.6689004	574.5878506	633.5066965
M18.01	Tepic	97	0.034730237	5939.134888	6617.724565	7296.313041	206.2675602	229.8351402	253.4026786
M18.01	Tepic	98	0.052095355	5939.134888	6617.724565	7296.313041	309.4013402	344.7527103	380.1040179
M18.01	Tepic	99	0.017365118	5939.134888	6617.724565	7296.313041	103.1337801	114.9175701	126.7013393
M18.01	Tepic	100	0.017365118	5939.134888	6617.724565	7296.313041	103.1337801	114.9175701	126.7013393
M18.01	Tepic	101	0.017365118	5939.134888	6617.724565	7296.313041	103.1337801	114.9175701	126.7013393
M18.01	Tepic	102	0.017365118	5939.134888	6617.724565	7296.313041	103.1337801	114.9175701	126.7013393
M18.01	Tepic	104	0.017365118	5939.134888	6617.724565	7296.313041	103.1337801	114.9175701	126.7013393
M19.01	Monterrey	30	0.061488303	264984.9071	273569.2702	282153.6204	16293.47215	16821.31007	17349.14719
M19.01	Monterrey	31	0.056040547	262984.079	271607.8344	280231.5766	14737.77175	15221.05172	15704.33095
M19.01	Monterrey	32	0.104942976	260904.6486	269568.2557	278231.8492	27380.11027	28289.29499	29198.47827
M19.01	Monterrey	33	0.083902241	258351.8207	267042.0411	275732.2474	21676.29679	22405.42575	23134.55354
M19.01	Monterrey	34	0.110675108	255829.4464	264556.3586	273283.2575	28313.95155	29279.80349	30245.65396
M19.01	Monterrey	35	0.128717901	253146.9684	261898.4041	270649.8262	32584.5463	33711.01273	34837.4774
M19.01	Monterrey	36	0.055498635	250281.0247	258930.9684	267580.8992	13890.25531	14370.31538	14850.37473
M19.01	Monterrey	37	0.022135022	247193.3706	255859.9456	264526.5073	5471.630585	5663.465409	5855.29994
M19.01	Monterrey	38	0.022135022	244191.2813	252867.6273	261543.9585	5405.179274	5597.23038	5789.281157
M19.01	Monterrey	39	0.112851463	240615.602	249290.476	257965.3347	27153.82279	28132.79502	29111.76552
M19.01	Monterrey	40	0.054404348	248319.1597	257396.3906	266473.6045	13509.6421	14003.48293	14497.32284

City ID	City name	Age	Avoidable deaths from respiratory causes mean estimate	Lower value, lost productivity	Center value, lost productivity	Upper value, lost productivity	Lost productivity by age, lower value	Lost productivity by age, center value	Lost productivity by age, upper value
M19.01	Monterrey	41	0.038543371	244541.9912	253490.9252	262439.8418	9425.472646	9770.394729	10115.31614
M19.01	Monterrey	42	0.14707861	240859.2752	249863.6964	258868.0982	35425.24745	36749.6052	38073.96011
M19.01	Monterrey	43	0.214211339	237075.8922	246130.5807	255185.2507	50784.34442	52723.96139	54663.57436
M19.01	Monterrey	44	0.21376055	232791.7506	241892.7143	250993.6576	49761.69265	51707.11966	53652.5423
M19.01	Monterrey	45	0.194352597	228437.6164	237566.9362	246696.235	44397.4439	46171.75092	47946.05385
M19.01	Monterrey	46	0.16152914	223786.1812	232955.8939	242125.585	36147.98929	37629.1651	39110.33741
M19.01	Monterrey	47	0.116992397	218892.778	228094.6904	237296.5806	25608.79087	26685.34466	27761.89585
M19.01	Monterrey	48	0.203852282	214115.6684	223327.584	232539.4754	43647.96755	45525.83757	47403.70265
M19.01	Monterrey	49	0.136396933	208604.7119	217706.1707	226807.6022	28453.04296	29694.45403	30935.86136
M19.01	Monterrey	50	0.275529812	203205.9144	212320.7746	221435.6054	55989.28731	58500.70303	61012.11063
M19.01	Monterrey	51	0.197023458	197480.3685	206319.2217	215158.0507	38908.26511	40649.72653	42391.18319
M19.01	Monterrey	52	0.16126244	192075.4023	200889.8926	209704.3581	30974.54812	32395.99435	33817.43657
M19.01	Monterrey	53	0.263244719	185909.9032	194679.321	203448.7168	48939.8002	51248.30312	53556.80027
M19.01	Monterrey	54	0.158028285	179215.3544	187637.6676	196059.9692	28321.09516	29652.05886	30983.02075
M19.01	Monterrey	55	0.410013926	172435.3946	180801.7815	189168.1577	70700.91316	74131.24826	77561.57902
M19.01	Monterrey	56	0.298201286	165344.1369	173545.9158	181747.6888	49305.83421	51751.61523	54197.39448
M19.01	Monterrey	57	0.188624169	158387.5396	166472.7814	174558.0232	29875.71802	31400.79003	32925.86206
M19.01	Monterrey	58	0.464784777	150533.8041	157853.3711	165172.9382	69965.82049	73367.84382	76769.86717
M19.01	Monterrey	59	0.358605764	143323.6936	150380.5496	157437.4095	51396.70265	53927.33192	56457.96255
M19.01	Monterrey	60	0.258075881	136365.6718	143188.0566	150010.4453	35192.69085	36953.38382	38714.0778
M19.01	Monterrey	61	0.325296315	128840.4808	135470.7378	142100.9927	41911.33365	44068.13183	46224.92931
M19.01	Monterrey	62	0.589834582	121032.942	127473.154	133913.3662	71389.41469	75188.07444	78986.73432

City ID	City name	Age	Avoidable deaths from respiratory causes mean estimate	Lower value, lost productivity	Center value, lost productivity	Upper value, lost productivity	Lost productivity by age, lower value	Lost productivity by age, center value	Lost productivity by age, upper value
M19.01	Monterrey	63	0.47865739	113724.5373	119961.2342	126197.9302	54435.09019	57420.33126	60405.57186
M19.01	Monterrey	64	0.397977086	106255.6606	112286.4575	118317.2546	42287.31815	44687.43712	47087.55617
M19.01	Monterrey	65	0.409516836	98876.48955	104605.7504	110335.0114	40491.5872	42837.81595	45184.0448
M19.01	Monterrey	66	0.302067007	91036.47528	96450.45266	101864.429	27499.1156	29134.49954	30769.88317
M19.01	Monterrey	67	0.53492381	82875.99698	87985.34363	93094.68925	44332.34406	47065.45524	49798.56586
M19.01	Monterrey	68	0.528788733	74954.00784	79625.54718	84297.08545	39634.83486	42105.09222	44575.34903
M19.01	Monterrey	69	0.644272	67038.25876	71179.86379	75321.46772	43190.87303	45859.19318	48527.51263
M19.01	Monterrey	70	0.687955478	59120.71311	62886.25236	66651.79047	40672.41845	43262.94181	45853.46438
M19.01	Monterrey	71	0.640131159	51361.54866	54689.02429	58016.49875	32878.12767	35008.1485	37138.16858
M19.01	Monterrey	72	0.6879166	43195.5088	46150.86938	49106.22876	29714.90757	31747.94917	33780.98994
M19.01	Monterrey	73	0.722124949	34718.40028	37278.76072	39839.12115	25071.02305	26919.9232	28768.82335
M19.01	Monterrey	74	0.538734323	26371.27699	28411.02289	30450.7688	14207.11204	15305.99317	16404.8743
M19.01	Monterrey	75	0.949728341	17201.3844	18613.94174	20026.49908	16336.64226	17678.188	19019.73374
M19.01	Monterrey	76	0.869535436	8262.323036	9031.581147	9800.839258	7184.38266	7853.279847	8522.177034
M19.01	Monterrey	77	0.763236097	8262.323036	9031.581147	9800.839258	6306.103187	6893.228745	7480.354304
M19.01	Monterrey	78	1.078297821	8262.323036	9031.581147	9800.839258	8909.244923	9738.734268	10568.22361
M19.01	Monterrey	79	1.100477001	8262.323036	9031.581147	9800.839258	9092.496473	9939.047332	10785.59819
M19.01	Monterrey	80	1.079029195	8262.323036	9031.581147	9800.839258	8915.287773	9745.339733	10575.39169
M19.01	Monterrey	81	1.085393071	8262.323036	9031.581147	9800.839258	8967.868175	9802.815599	10637.76302
M19.01	Monterrey	82	1.120659041	8262.323036	9031.581147	9800.839258	9259.24701	10121.32307	10983.39912
M19.01	Monterrey	83	1.075257467	8262.323036	9031.581147	9800.839258	8884.124537	9711.275065	10538.42559
M19.01	Monterrey	84	1.327321921	8262.323036	9031.581147	9800.839258	10966.76249	11987.81564	13008.86879

City ID	City name	Age	Avoidable deaths from respiratory causes mean estimate	Lower value, lost productivity	Center value, lost productivity	Upper value, lost productivity	Lost productivity by age, lower value	Lost productivity by age, center value	Lost productivity by age, upper value
M19.01	Monterrey	85	0.873845744	8262.323036	9031.581147	9800.839258	7219.995818	7892.208745	8564.421671
M19.01	Monterrey	86	1.113883826	8262.323036	9031.581147	9800.839258	9203.267995	10060.13216	10916.99633
M19.01	Monterrey	87	0.863499103	8262.323036	9031.581147	9800.839258	7134.508528	7798.762216	8463.015905
M19.01	Monterrey	88	0.753051331	8262.323036	9031.581147	9800.839258	6221.95336	6801.244204	7380.535049
M19.01	Monterrey	89	0.686873696	8262.323036	9031.581147	9800.839258	5675.17236	6203.555522	6731.938683
M19.01	Monterrey	90	0.694507427	8262.323036	9031.581147	9800.839258	5738.244709	6272.50018	6806.755651
M19.01	Monterrey	91	0.887272907	8262.323036	9031.581147	9800.839258	7330.935378	8013.477258	8696.019139
M19.01	Monterrey	92	0.790959339	8262.323036	9031.581147	9800.839258	6535.161565	7143.613452	7752.065339
M19.01	Monterrey	93	0.513917236	8262.323036	9031.581147	9800.839258	4246.15022	4641.485223	5036.820225
M19.01	Monterrey	94	0.631606013	8262.323036	9031.581147	9800.839258	5218.53291	5704.400959	6190.269007
M19.01	Monterrey	95	0.40316064	8262.323036	9031.581147	9800.839258	3331.043445	3641.178037	3951.31263
M19.01	Monterrey	96	0.404029253	8262.323036	9031.581147	9800.839258	3338.220204	3649.022984	3959.825764
M19.01	Monterrey	97	0.127083457	8262.323036	9031.581147	9800.839258	1050.004574	1147.764554	1245.524534
M19.01	Monterrey	98	0.168087937	8262.323036	9031.581147	9800.839258	1388.796838	1518.099847	1647.402856
M19.01	Monterrey	99	0.1219037	8262.323036	9031.581147	9800.839258	1007.207748	1100.983158	1194.758568
M19.01	Monterrey	100	0.175769554	8262.323036	9031.581147	9800.839258	1452.264834	1587.476989	1722.689144
M19.01	Monterrey	101	0.138312049	8262.323036	9031.581147	9800.839258	1142.77883	1249.176496	1355.574162
M19.01	Monterrey	102	0.104948435	8262.323036	9031.581147	9800.839258	867.1178759	947.8503111	1028.582746
M19.01	Monterrey	104	0.059319069	8262.323036	9031.581147	9800.839258	490.1133073	535.744982	581.3766566
M19.01	Monterrey	105	0.016408349	8262.323036	9031.581147	9800.839258	135.5710821	148.1933379	160.8155936
M19.01	Monterrey	108	0.022135022	8262.323036	9031.581147	9800.839258	182.8866984	199.9142433	216.9417882
M30.08	Xalapa	32	0.012297348	161083.4463	201346.6318	242577.8204	1980.899125	2476.029511	2983.063767

City ID	City name	Age	Avoidable deaths from respiratory causes mean estimate	Lower value, lost productivity	Center value, lost productivity	Upper value, lost productivity	Lost productivity by age, lower value	Lost productivity by age, center value	Lost productivity by age, upper value
M30.08	Xalapa	34	0.012297348	154347.228	195374.5401	237428.804	1898.061508	2402.588623	2919.744524
M30.08	Xalapa	36	0.012297348	152476.4821	193888.3512	236389.7137	1875.056295	2384.312443	2906.966469
M30.08	Xalapa	38	0.012297348	146486.4001	186229.1416	227127.7255	1801.394174	2290.124481	2793.068581
M30.08	Xalapa	39	0.012297348	145501.9402	184531.6472	224751.8729	1789.28793	2269.249801	2763.851896
M30.08	Xalapa	42	0.024594695	144087.8193	183811.5075	224836.1078	3543.795985	4520.787987	5529.775523
M30.08	Xalapa	49	0.012297348	122693.5564	160715.3789	200337.1565	1508.805306	1976.372873	2463.615643
M30.08	Xalapa	50	0.012297348	118931.8529	156288.1382	195292.3774	1462.546331	1921.929555	2401.57824
M30.08	Xalapa	52	0.024594695	111096.7082	148578.135	187807.8785	2732.389667	3654.233933	4619.077513
M30.08	Xalapa	58	0.024594695	80228.29494	114648.8739	151157.0261	1973.190454	2819.7541	3717.660971
M30.08	Xalapa	59	0.024594695	77026.42827	109271.7941	143667.3603	1894.44152	2687.506461	3533.454924
M30.08	Xalapa	60	0.024594695	74358.82377	104875.7482	137607.3777	1828.8326	2579.387052	3384.411502
M30.08	Xalapa	62	0.036892043	64601.58099	93360.88738	124469.7745	2383.284283	3444.273842	4591.944232
M30.08	Xalapa	63	0.012297348	59146.50819	86827.85596	116929.2718	727.3451682	1067.752323	1437.919896
M30.08	Xalapa	65	0.012297348	48651.79467	73629.43429	101174.5241	598.2880285	905.4467441	1244.178287
M30.08	Xalapa	66	0.024594695	44946.46857	68394.88939	94487.78518	1105.444691	1682.151452	2323.898269
M30.08	Xalapa	67	0.04918939	35420.25472	57929.16611	83161.88919	1742.300732	2849.500358	4090.682621
M30.08	Xalapa	68	0.073784085	30216.18256	50043.79068	72676.92733	2229.473393	3692.435324	5362.40061
M30.08	Xalapa	70	0.04918939	24881.13695	37621.80469	53338.86134	1223.887955	1850.593632	2623.706066
M30.08	Xalapa	71	0.09837878	23036.92705	33091.89572	46212.54373	2266.344789	3255.540345	4546.333695
M30.08	Xalapa	72	0.036892043	21137.39085	28426.08947	38872.43658	779.8015255	1048.696506	1434.083589
M30.08	Xalapa	73	0.04918939	17808.25137	22963.50369	31371.13394	875.9770262	1129.560744	1543.12695
M30.08	Xalapa	74	0.09837878	12528.43386	15897.97946	22617.47434	1232.532044	1564.023831	2225.079543

City ID	City name	Age	Avoidable deaths from respiratory causes mean estimate	Lower value, lost productivity	Center value, lost productivity	Upper value, lost productivity	Lost productivity by age, lower value	Lost productivity by age, center value	Lost productivity by age, upper value
M30.08	Xalapa	75	0.036892043	7090.221814	10345.71456	13601.20495	261.5727658	381.6745432	501.7762334
M30.08	Xalapa	76	0.061486738	2412.661759	4440.788727	6468.914495	148.346701	273.0496121	397.7524494
M30.08	Xalapa	77	0.073784085	2412.661759	4440.788727	6468.914495	178.0160412	327.6595345	477.3029393
M30.08	Xalapa	78	0.073784085	2412.661759	4440.788727	6468.914495	178.0160412	327.6595345	477.3029393
M30.08	Xalapa	79	0.086081433	2412.661759	4440.788727	6468.914495	207.6853814	382.2694569	556.8534291
M30.08	Xalapa	80	0.086081433	2412.661759	4440.788727	6468.914495	207.6853814	382.2694569	556.8534291
M30.08	Xalapa	81	0.073784085	2412.661759	4440.788727	6468.914495	178.0160412	327.6595345	477.3029393
M30.08	Xalapa	82	0.086081433	2412.661759	4440.788727	6468.914495	207.6853814	382.2694569	556.8534291
M30.08	Xalapa	83	0.122973476	2412.661759	4440.788727	6468.914495	296.6934019	546.0992242	795.5048988
M30.08	Xalapa	84	0.09837878	2412.661759	4440.788727	6468.914495	237.3547216	436.8793793	636.403919
M30.08	Xalapa	85	0.135270823	2412.661759	4440.788727	6468.914495	326.3627421	600.7091466	875.0553886
M30.08	Xalapa	86	0.061486738	2412.661759	4440.788727	6468.914495	148.346701	273.0496121	397.7524494
M30.08	Xalapa	87	0.073784085	2412.661759	4440.788727	6468.914495	178.0160412	327.6595345	477.3029393
M30.08	Xalapa	88	0.09837878	2412.661759	4440.788727	6468.914495	237.3547216	436.8793793	636.403919
M30.08	Xalapa	89	0.086081433	2412.661759	4440.788727	6468.914495	207.6853814	382.2694569	556.8534291
M30.08	Xalapa	90	0.061486738	2412.661759	4440.788727	6468.914495	148.346701	273.0496121	397.7524494
M30.08	Xalapa	91	0.061486738	2412.661759	4440.788727	6468.914495	148.346701	273.0496121	397.7524494
M30.08	Xalapa	92	0.061486738	2412.661759	4440.788727	6468.914495	148.346701	273.0496121	397.7524494
M30.08	Xalapa	93	0.036892043	2412.661759	4440.788727	6468.914495	89.00802058	163.8297673	238.6514696
M30.08	Xalapa	94	0.036892043	2412.661759	4440.788727	6468.914495	89.00802058	163.8297673	238.6514696
M30.08	Xalapa	95	0.04918939	2412.661759	4440.788727	6468.914495	118.6773608	218.4396897	318.2019595
M30.08	Xalapa	96	0.04918939	2412.661759	4440.788727	6468.914495	118.6773608	218.4396897	318.2019595

City ID	City name	Age	Avoidable deaths from respiratory causes mean estimate	Lower value, lost productivity	Center value, lost productivity	Upper value, lost productivity	Lost productivity by age, lower value	Lost productivity by age, center value	Lost productivity by age, upper value
M30.08	Xalapa	97	0.024594695	2412.661759	4440.788727	6468.914495	59.33868039	109.2198448	159.1009798
M30.08	Xalapa	98	0.024594695	2412.661759	4440.788727	6468.914495	59.33868039	109.2198448	159.1009798
M30.08	Xalapa	99	0.012297348	2412.661759	4440.788727	6468.914495	29.66934019	54.60992242	79.55048988
M30.08	Xalapa	101	0.012297348	2412.661759	4440.788727	6468.914495	29.66934019	54.60992242	79.55048988
